# Core cysteine residues in the Plasminogen-Apple-Nematode (PAN) domain are critical for HGF/c-MET signaling

**DOI:** 10.1038/s42003-022-03582-8

**Published:** 2022-07-01

**Authors:** Debjani Pal, Kuntal De, Carly M. Shanks, Kai Feng, Timothy B. Yates, Jennifer Morrell-Falvey, Russell B. Davidson, Jerry M. Parks, Wellington Muchero

**Affiliations:** 1 Bioscience Division, Oak Ridge National Laboratory, 1 Bethel Valley Rd, Oak Ridge, TN 37831 USA; 2 Bredesen Center for Interdisciplinary Research, University of Tennessee, Knoxville, TN 37996 USA

**Keywords:** Cell signalling, Proteins

## Abstract

The Plasminogen-Apple-Nematode (PAN) domain, with a core of four to six cysteine residues, is found in > 28,000 proteins across 959 genera. Still, its role in protein function is not fully understood. The PAN domain was initially characterized in numerous proteins, including HGF. Dysregulation of HGF-mediated signaling results in multiple deadly cancers. The binding of HGF to its cell surface receptor, c-MET, triggers all biological impacts. Here, we show that mutating four core cysteine residues in the HGF PAN domain reduces c-MET interaction, subsequent c-MET autophosphorylation, and phosphorylation of its downstream targets, perinuclear localization, cellular internalization of HGF, and its receptor, c-MET, and c-MET ubiquitination. Furthermore, transcriptional activation of HGF/c-MET signaling-related genes involved in cancer progression, invasion, metastasis, and cell survival were impaired. Thus, targeting the PAN domain of HGF may represent a mechanism for selectively regulating the binding and activation of the c-MET pathway.

## Abstract (web summary)

Core cysteine residues in the Plasminogen-Apple-Nematode (PAN) domain are essential for HGF/c-MET signaling and regulate their interaction, perinuclear localization, and cellular internalization. Thus, targeting PAN may represent a mechanism for the selective regulation of the c-MET pathway, with relevance to cancer treatment.

## Introduction

Despite its prevalence in a wide variety of organisms, the role of the Plasminogen-Apple-Nematode (PAN) domain in protein function has largely remained elusive. A key contributing factor is that this domain is found in many phylogenetically unrelated proteins across divergent organisms falling into categories including Alveolata, Archea, Amoebazoa, Bacteria, Cryptophyta, Euglenozoa, Haptophyceae, Opisthokonta, Rhizaria, Rhodophyta, Stramenopilles, Viridplantae, and Viruses. The PAN domain was first identified by Tordai et al.^1^ in which they noted that the domain was shared by the plasminogen/hepatocyte growth factor (HGF) protein family, the prekallikrein/coagulation factor XI protein family, and nematode proteins. The domain possesses the characteristic 4-6-cysteine residues in its core that are strictly conserved. These cysteine residues have been proposed to engage in two or three disulfide bridges to form a hairpin loop structure^1,2^. The PAN domain has been suggested to mediate protein-protein or carbohydrate-protein interactions and facilitate receptor dimerization^2–6^. HGF is secreted by mesenchymal cells as a single-chain, biologically inert precursor and is converted into its bioactive form when extracellular proteases cleave the bond between Arg494 and Val495^7^. The mature form of HGF consists of an α- and β-chain, which are held together by a disulfide bond^8–10^. The α-subunit of HGF includes N-terminal hairpin loop structure and four kringle domains (K1-K4) whereas the β-subunit consists of serine protease homology (SPH) domain^11^.

To provide evidence for its role in cellular mesenchymal-epidermal transition (c-MET) interaction and signal transduction, we characterized the functional role of the PAN domain in the heparin binding glycoprotein HGF, which functions as a ligand for the high-affinity receptor, c-MET. HGF/c-MET signaling has been shown to mediate cellular processes including angiogenesis, anti-apoptosis, mitogenesis, morphogenesis, mitogenesis and neurite extension^12^. Dysregulation of the HGF/c-MET signaling cascade can lead to tumorigenesis by transforming normal cells into tumor cells and c-MET hyperactivation has been reported in cancers including lung cancer, colorectal cancer, glioblastoma, and acute myeloid lymphoma among others^13,14^.

A crucial step in HGF/c-MET cascade activation is that the binding of HGF to c-MET brings the dimerization of c-MET that enables its intracellular kinase domains to undergo autophosphorylation^14^. The phosphorylated kinase domain recruits downstream cytosolic effector proteins, leading to the activation of downstream signaling pathways.

The HGF/c-MET pathway has emerged as a prime target for cancer pathways and tumorigenesis^15^ However, despite the clinical therapeutic significance of this pathway, the mechanism by which HGF activates c-MET is not well understood. HGF is a bivalent ligand with high-affinity binding pockets in the α chain and relatively low-affinity binding pockets in the β chain^16^. Studies demonstrated that the N- domain and the first kringle domain are enough for c-MET binding; however, for activation of c-MET, the β chain is essential^16^. Although the affinity of the α chain of HGF and the SEMA domain of MET is structurally well-characterized and functionally validated, it is not clear what regions of the α chain bind with c-MET.

## Results

### PAN domain-carrying proteins are enriched in cell recognition and cellular signaling processes

PAN domain-carrying proteins do not share a clear evolutionary or phylogenetic trajectory suggesting that this domain may have been co-opted independently by organisms to serve protein functions that are yet to be revealed. To provide evidence of its putative function, we scanned the InterPro (https://www.ebi.ac.uk/interpro/) and UniProt (https://G-LecRK.uniprot.org) databases and found 28,300 proteins across 2496 organisms falling into 959 genera (Supplementary Data 1). Gene Ontology (GO) enrichment analyses using the 28,300 proteins revealed 39 unique GO-terms that were enriched at *p* < 0.05 (Supplementary Data 2). These included terms such as cell recognition (*p-*value = 1E−30), cell communication (*p-*value = 1E−30), proteolysis (*p-*value = 1E−30), pollen-pistil interaction (*p-*value = 1E−30), reproduction (*p-*value = 1E−30), response to stimulus (*p-*value = 1E−30) and response to stress (*p-*value = 1E−30). Interestingly, the PAN domain resembles striking similarities with cysteine-rich peptides (CRPs) that have been widely implicated in the regulation of immune responses^17–23^. Based on a GO enrichment analysis and predicted cellular localization involving 28,000 PAN domain-containing proteins, we found that these proteins were highly enriched in protein proteolysis and immune signaling processes. Almost all of them were associated with the extracellular matrix (ECM) as cell surface receptors or as ligands for cell surface receptors and typically associated with cellular signal transduction, albeit in different pathways for divergent organisms. We therefore hypothesized that the HGF PAN domain would be essential for HGF and c-MET interaction and the downstream signaling. To test this possibility, we designed experiments to mutate core cysteine residues and assess implications on downstream signaling cascades.

### Core PAN domain cysteine residues modulate HGF overall-abundance

**Fig. 1:**
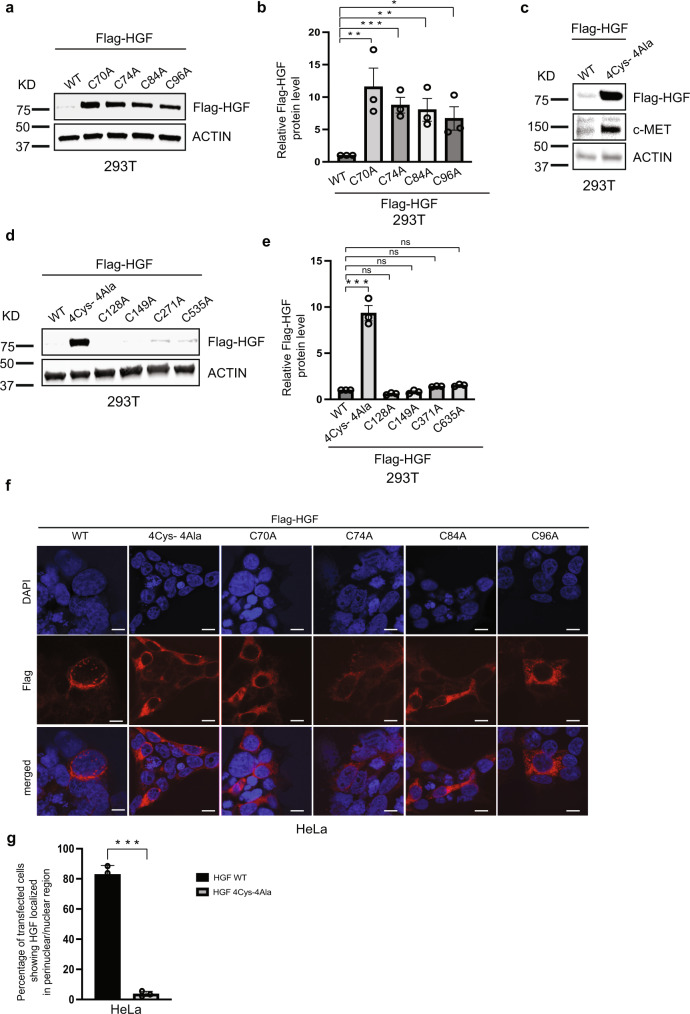
PAN domain modulates HGF stability. **a**, **b** Core cysteines in PAN domain are crucial for HGF stability. **a** Immunoblot analysis of whole cell lysates derived from 293T cells, transfected with Flag-HGF WT and different single cysteine mutants of Flag-HGF constructs as indicated. 30 h post-transfection, whole-cell lysates were prepared for immunoblot analysis. Representative image of *n* = 3 biological replicates. **b** Quantification of the band intensities in (**a**). The intensities of Flag-HGF (WT and mutants) bands were normalized to actin and then normalized to Flag-HGF WT. Data are represented as mean ± SEM, *n* = 3, and *p < 0.05, **p < 0.005, *** *p* < 0.0005 (student’s t-test). **c** Mutation in all four core cysteine residues in PAN domain remarkably alters HGF stability. Immunoblot analysis of whole cell lysates derived from 293 T cells, transfected with Flag-HGF WT and Flag-HGF 4Cys-4Ala constructs as indicated. 30 h post-transfection, whole-cell lysates were prepared for immunoblot analysis. **d** Cysteines in kringle domains and SPH domain of HGF have no impact on the protein abundance in cells. Immunoblot analysis of whole cell lysates derived from 293T cells, transfected with Flag-HGF WT, Flag-HGF 4Cys-4Ala, and different single cysteine mutants of Flag-HGF constructs as indicated. 30 h post-transfection, whole-cell lysates were prepared for immunoblot analysis. Representative image of *n* = 3 biological replicates. **e** Quantification of the band intensities in (**d**). The intensities of Flag-HGF (WT and mutants) bands were normalized to actin and then normalized to Flag-HGF WT. Data are represented as mean ± SEM, *n* = 3, and *p < 0.05, **p < 0.005, ***p < 0.0005 (student’s t-test). **f** Localization of Flag-HGF WT and different Flag-HGF mutants’ expression by confocal immunofluorescence microscopy in HeLa cells. The cells were transiently transfected with Flag-HGF WT and different mutants of Flag-HGFs as indicated. 30 h post-transfection cells were fixed, mounted and protein expression patterns were visualized using a Zeiss LSM 710 confocal microscope outfitted with a 63× objective. Scale bars represent 20 μm. The images shown are representative from three independent biological experiments (average 100 cells were observed per experimental condition per replicate). **g** Percentage of transfected HeLa cells showing perinuclear/nuclear staining for Flag-HGF WT and Flag-HGF 4Cys-4Ala were quantified. Data are represented as mean ± SD, *n* = 3 (average 100 cells were observed for each condition per experiment), and *p < 0.05, **p < 0.005, *** *p* < 0.0005 (Student’s t test).

Alignment of the PAN domain of proteins from 14 model organisms revealed four strictly conserved cysteine residues occurring at amino acid positions 70, 74, 84, and 96 of the HGF protein (Supplementary Figs. 1a, b, 2a–c). To evaluate the functional significance of these residues, we sequentially mutated single cysteines and simultaneously mutated all four cysteine residues and examined HGF stability in 293T cells. Mutant HGFs, in which cysteines 70 (C70A), 74 (C74A), 84 (C84A), and 96 (C96A) were substituted with alanine, led to a marked increase in the protein expression of exogenously expressed HGFs in cells compared to their wild-type (WT) counterpart (Fig. 1a, b). Furthermore, mutating all four core cysteines in the PAN domain dramatically increased the abundance of exogenously expressed HGF-4Cys-4Ala in the cells (Fig. 1c). Moreover, the c-MET receptor exhibited a similar increase in abundance in response to stimulation by the HGF 4Cys-4Ala mutant compared to WT HGF. These results suggest that mutant HGF and its c-MET receptor did not undergo the expected downregulation that was previously reported via ligand-induced internalization following ubiquitination and lysosomal degradation^24^.

The kringle domains of HGF have been reported to be crucial for protein-protein interactions. In particular, the first and second kringle domains are especially important for the proper biological function of the protein^11^. Kringle domains are usually disulfide crosslinked domain and previous studies have established that kringle domains in the α-subunit and the SPH domain in the β-subunit provide c-MET binding sites on HGF^25,26^. Recently, Uchikawa et al., determined structures of c-MET/HGF complex mimicking their active state at 4.8 Å resolution using cryo-electron microscopy^26^. The study identified multiple distinctive c-MET binding sites on the HGF protein including the N-terminal and kringle domains but did not implicate the PAN domain in the HGF/c-MET interaction^26^. Here, we exclusively investigated the role of the PAN domain of HGF on the c-MET binding and downstream signaling cascade.

As we established a connection between the core cysteines in the PAN domain of HGF with HGF abundance, we introduced four more mutations of additional cysteines located in the K1, K2, and SPH domains of HGF (C128A, C149A, C271A, and C535A). None of these mutant HGFs showed increased protein expression when expressed exogenously in cells as did the HGF 4Cys-4Ala (Fig. 1d, e). Thus, cysteine residues in the kringle and SPH domains are apparently not involved in HGF abundance. Rather, induced expression of HGF is specifically defined by the four strictly conserved cysteine residues in PAN domain.

We hypothesized that the enhanced HGF expression of the 4Cys-4Ala mutant may results from an intra-PAN domain structural change that prevents HGF degradation. To test this hypothesis, we performed molecular dynamics (MD) simulations of the WT and 4Cys-4Ala PAN domains using models generated with AlphaFold2^27^ and compared the resulting structural ensembles. Five models were generated for each system and used as starting structures for the simulations. A 200 ns simulation was then performed for each model. Thus, the cumulative simulation time was 1 μs for each system. Analysis of the time evolution of the root-mean-square deviation (RMSD) of each system revealed only minor structural deviations in both sets of simulations. Using the top WT model as a reference structure, the average RMSDs and standard errors of the means were 1.61 +/− 0.02 Å for the WT and 1.71 +/− 0.01 Å for the 4Cys-4Ala system, indicating highly similar overall structures despite the four cysteine substitutions in the mutant (Supplementary Fig. 3a, b). The structural analysis indicates that no large-scale structural changes occurred in the 4Cys-4Ala mutant PAN domain compared to the WT on this time scale (Supplementary Fig. 4).

Upon binding with its high-affinity protooncogenic receptor, c-MET, HGF activates a wide range of cellular signaling pathways. Autophosphorylation in the carboxy-terminal tail of c-MET creates a binding sites for more downstream adaptors^7^. However, the immediate removal of the ligand-bound receptor from the plasma membrane is essential to impede the sustained stimulation of the receptor and is tightly controlled by the rapid internalization and degradation of the ligand and degradation/recycling of the receptors^28^. Translocation of c-MET-bound HGF toward the perinuclear region is a pivotal part of c-MET endocytosis^7,24,28,29^. The existence of a parallel pathway has also been reported in which HGF-activated c-MET translocates to the nucleus to initiate calcium signaling^30^. In agreement with a role for the PAN domain in HGF abundance, we found that mutating the core cysteine residues reduced the perinuclear signal for all mutated HGFs in HeLa cells (Fig. 1f). HeLa cells transfected with HGF 4Cys-4Ala showed less than 5% of the total perinuclear staining of recombinant HGF. However, under the same transfection efficiency, 80% of cells transfected with WT HGF showed perinuclear staining for HGF under confocal microscopy (Fig. 1g). As such, both biochemical and immunofluorescence results suggest a critical role for core PAN domain cysteine residues in HGF stability and cellular uptake. Because all single cysteine mutants yielded the same results as the 4Cys-4Ala mutant, all subsequent studies were performed using only the recombinant HGF 4Cys-4Ala variant.

### PAN domain cysteine residues are essential for c-MET, AKT, and ERK phosphorylation

**Fig. 2:**
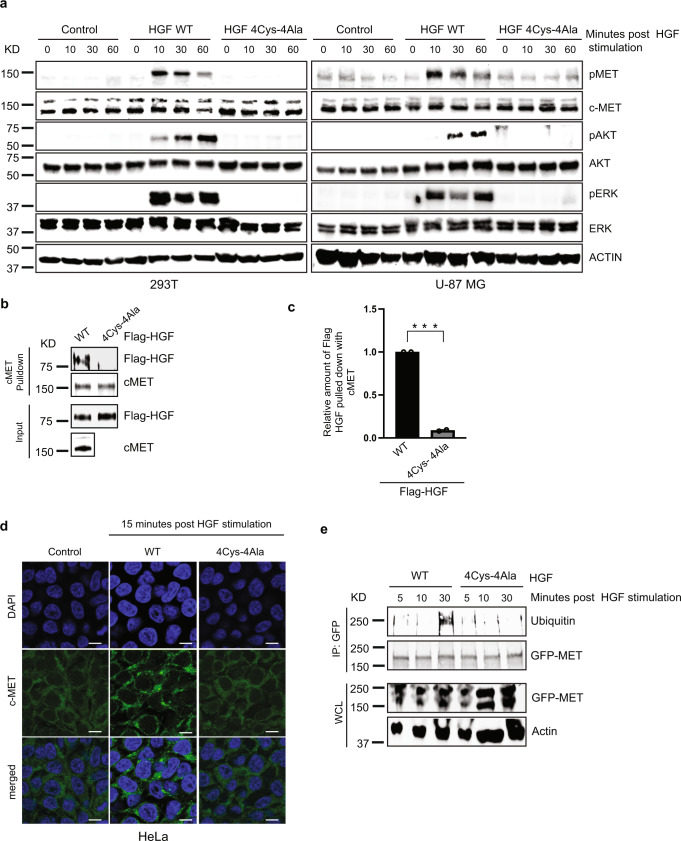
HGF PAN domain regulates c-MET signaling cascade via four core cysteines. **a** Mutation of the core cysteines in HGF PAN domain blocks HGF-induced c-MET signaling. Both 293T and U-87 MG cells were stimulated with HGF WT and HGF 4Cys-4Ala proteins for the indicated amount of time. Cells were harvested and immunoblot analysis shows the absence of phosphorylation for c-MET, AKT, and ERK in presence of HGF 4Cys-4Ala. Representative blot images from *n* = 2 experiments for individual cell line. **b** Immunoblot showing that the core cysteines on the HGF PAN domain regulate its binding with c-MET. c-MET was immunoprecipitated from 293T cells on anti-MET bound beads. In-vitro translated Flag-tagged HGF WT and HGF 4Cys-4Ala were added to the beads as indicated to detect the interaction between endogenous c-MET and Flag-HGF WT and Flag-HGF 4Cys-4Ala. **c** Right panel, quantification of the band intensities (*n* = 2; *** *p* < 0.0005 (Student’s t test)). Immunoprecipitated Flag-HGFs band intensities were normalized to the respective c-MET IP bands and then further normalized to HGF-WT. **d** HGF PAN domain defines perinuclear translocation of c-MET in cells. HeLa cells were stimulated with HGF WT and HGF 4Cys-4Ala proteins for the indicated amount of time following serum starvation. Post stimulation cells were fixed, mounted and endogenous c-MET localization pattern was visualized using Zeiss LSM 710 at 63x objective. Scale bars represent 20 μm. The images shown are representative from three independent biological replicates (average 100 cells were observed for each condition per replicate). **e** PAN domain regulates c-MET ubiquitination. In vivo ubiquitination assay shows that HGF WT promotes c-MET ubiquitination in a PAN-dependent manner. 293T cells were transfected with the construct c-MET-C-GFPSpark. After serum starvation, cells were stimulated with HGF WT and HGF 4Cys-4Ala as indicated. The lysates were collected at specific time points and incubated with anti-GFP protein G beads. Ubiquitinated-c-MET proteins were eluted, resolved by SDS-PAGE, and immunoblotted with the indicated antibodies.

Following binding of HGF at the MET semaphoring homology (SEMA) domain, c-MET homodimerizes and autophosphorylates at two tyrosine residues (Y1234 and Y1235), followed by subsequent phosphorylation of two additional tyrosines in the carboxy-terminal tail (Y1349 and Y1356)^31^. This series of events creates a multifunctional docking site for downstream adaptors and effectors that lead to the activation of the Rat sarcoma (RAS)/extracellular signal-regulated kinase (ERK) and phosphatidylinositol 3-kinase (PI3K)/protein kinase B (AKT) axis via phosphorylation^32,33^. To determine the influence of core cysteines on c-MET phosphorylation, 293T, glioblastoma U-87 MG, and HeLa cells were stimulated with purified wild-type HGF and HGF 4Cys-4Ala proteins. Western blot analyses revealed the absence of phosphorylated c-MET, AKT, and ERK in unstimulated controls and in cells stimulated with the HGF 4Cys-4Ala protein in both cell types (Fig. 2a and Supplementary Fig. 5a, b). Additionally, purified proteins of HGF C70A, HGF C74A, HGF C84A, and HGF C96A mutants failed to promote downstream c-MET phosphorylation following stimulation as evident in Supplementary Fig. 6. This result suggests that each cysteine residues in the PAN HGF domain are critical for HGF/c-MET signaling in a wide variety of cells and thus indicates the potential for designing novel HGF specific inhibitor targeting dysregulated c-MET signaling cascade in pathological conditions.

To characterize the incompetence of PAN mutant HGF in turning on the phosphorylation cascade of c-MET, we performed a direct interaction assay between endogenous c-MET and in vitro translated HGF proteins and showed that HGF 4Cys-4Ala was unable to interact with c-MET (Fig. 2b, c). We further got interested in whether there was a complete lack of interaction between c-MET and PAN mutant HGF or whether it could not maintain stable interaction. We employed both in vitro and in vivo cross-linking approaches to demonstrate the interaction stability. Purified HGF WT protein and HGF 4Cys-4Ala protein were activated by incubation with biotin-containing trifunctional cross-linking reagent Sulfo-SBED (Thermo). This reagent has amine group-specific reactivity, a nonspecific photo reactivity, and a biotin as a reactive handle. The cross-linking can be reversed by thiol cleavage. Upon reduction of the disulfide bond, the biotin label is transferred to the interacting protein. Non-reacted crosslinker was removed by overnight dialysis. Cross-linking was induced by UV irradiation following incubation with purified c-MET. Biotin transfer from PAN mutant HGF to c-MET was reduced significantly as compared to HGF WT (Supplementary Fig. 7a, b). The result suggests that PAN mutant HGF makes direct contact with c-MET but unable to maintain stable interaction which attenuates downstream signaling.

As c-MET is an integral membrane protein, we used a whole-cell lysate crosslinking assay to demonstrate the significance of the PAN domain on c-MET interaction. We transfected HEK293T cells transfected with a vector expressing GFP-c-MET as target cells. The activation of HGF WT protein and HGF 4Cys-4Ala protein was done similarly as described above. Activated purified biotinylated HGF WT protein and HGF 4Cys-4Ala protein were incubated with GFP-c-MET expressing cell lysate in the dark for 45 min, and crosslinking was induced by UV irradiation. After crosslinking, biotin-containing complexes were immunoprecipitated on streptavidin beads and analyzed by Western blotting for the presence of GFP-c-MET. As shown in Supplementary Fig. 7c, PAN mutant HGF show substantially reduced interaction with c-MET, which bolstered our conclusion that the PAN domain of HGF plays a crucial role in maintaining stable interaction.

Our results thus far indicate a central role for the PAN domain in its initial recognition by c-MET. In addition, HGF 4Cys-4Ala, like the unstimulated controls, failed to promote c-MET perinuclear translocation compared to wild-type HGF based on stimulation assays using HeLa cells (Fig. 2d).

As our cross-linking experiment suggested a significantly reduced interaction of c-MET and HGF PAN mutant compared to its wild-type counterpart, we were interested in checking if that reduced interaction was sufficient to turn ON the ubiquitination status of c-MET. We performed an in-vivo ubiquitination assay with c-MET. Ubiquitination was inhibited with the alanine mutants of HGF compared to wild type (Fig. 2e). Based on these observations, we propose that due to the lack of activation, c-MET receptors are unable to become internalized by endocytosis, suggesting that the mutated cysteine residues impose an overall retardation of c-MET activity and its endocytic trafficking.

### Mutating core cysteine residues suppresses STAT3 phosphorylation and nuclear translocation

**Fig. 3:**
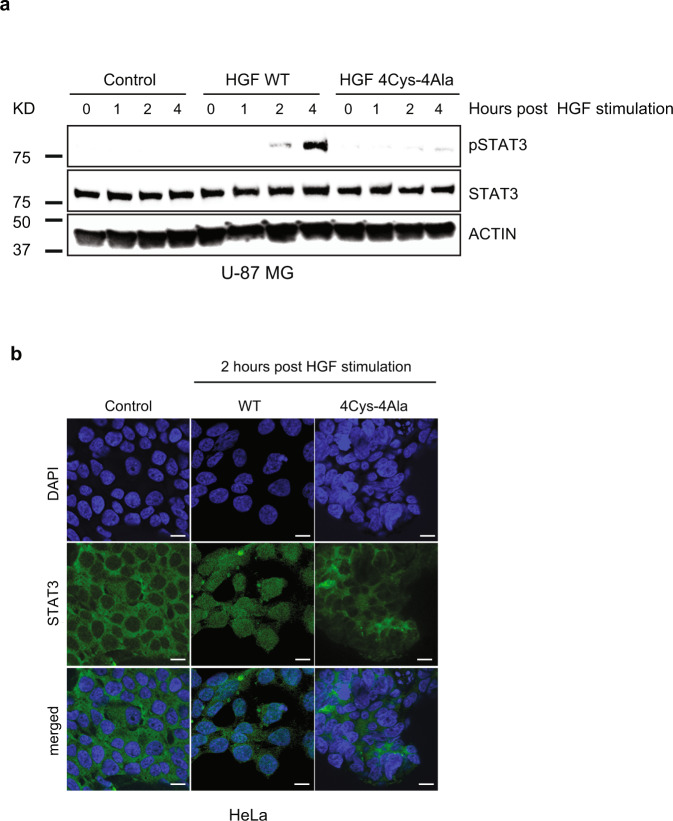
Core cysteines in HGF PAN domain is essential for STAT3 phosphorylation and nuclear translocation. **a** Impaired HGF PAN domain is unable to initiate STAT3 phosphorylation. U-87 MG cells were treated with HGF WT and HGF 4Cys-4Ala where indicated for 1, 2, and 4 h. Cell extracts were prepared and probed for phosphor-STAT3 and total STAT3. **b** STAT3 nuclear localization is suppressed by PAN mutant HGF. HeLa cells were stimulated with HGF WT and HGF 4Cys-4Ala as indicated. Cells were fixed and STAT3 was immunostained with a STAT3-specific antibody. The localization of STAT3 (green) and 4,6-diamidino-2-phenylindole (DAPI) (blue) in U-87 MG cells. Images were visualized using Zeiss LSM 710 at 63× objective. Scale bars represent 20 μm. The images shown are representative of three independent biological replicates (an average of 100 cells were observed for each condition per replicate).

signal transducer and activator of transcription 3 (STAT3) is a transcription factor that is present in the cytoplasm, forms dimers upon activation, and functions as a downstream effector molecule of the HGF/c-MET signaling pathway^34–36^. STAT3 is reported to be constitutively active in several cancers, which leads to malignant transformation by playing a critical role in stimulating cell proliferation and arresting apoptosis^36^. We tested the impact of the core cysteines in the HGF PAN domain on STAT3 activation by following its phosphorylation in glioblastoma U-87 MG and HeLa cells. STAT3 phosphorylation was notably reduced in cells post-stimulation with the HGF 4Cys-4Ala mutant compared to the wild type (Fig. 3a and Supplementary Fig. 8). Delayed time points were chosen for this assay because HGF induces delayed STAT3 phosphorylation^37^. Confocal imaging confirmed that HGF 4Cys-4Ala, like the unstimulated controls, was unable to promote STAT3 nuclear translocation, while wild-type HGF promoted normal STAT3 nuclear localization (Fig. 3b). These results are consistent with the above observations, suggesting that mutating the cysteine residues in the PAN domain leads to disrupted HGF/c-MET signaling.

### PAN mutations downregulate HGF/c-MET-dependent cell proliferation and alters the expression of genes essential for diverse cellular responses

Dysregulated expression of HGF/c-MET acts as a catalyst in many cancers, with overexpression of HGF often leading to aberrant cell proliferation and extracellular matrix invasion^14^. Direct evidence has been established connecting a primary role for HGF with increased expression of matrix metalloproteinase-9 (MMP9), which is crucial for angiogenesis^38^. HGF facilitates MMP9 expression via the PI3K/AKT and p38 mitogen-activated protein kinases (MAPK) axis. Given the reported role of HGF in modulating expression of MMP9, we evaluated the impact of mutated cysteine residues on its transcriptional response. To evaluate the role of PAN domain mutation on the pattern of the signature gene expression as well as cell proliferation, both 293T and U-87 MG cells were stimulated with wild-type HGF or 4Cys-4Ala HGF for 24 h following serum starvation. Cell viabilities, as assessed by the MTT assay, were notably reduced by treatment with HGF 4Cys-4Ala, whereas wild-type HGF could overcome serum starvation-mediated growth inhibition in both cell types (Supplementary Fig. 9a). 293T cells transiently transfected with Flag-WT HGF showed a significant increase in cell proliferation over time compared to cells transfected with Flag-HGF 4Cys-4Ala (Supplementary Fig. 10a). Quantitative real-time PCR (qRT-PCR) was performed to determine whether MMP9 expression was similarly impacted. mRNA levels of MMP9 were markedly decreased in HGF 4Cys-4Ala-stiumlated 293T and U-87 MG cells compared to the wild type in both cell types (left panel, Supplementary Fig. 9b). Similarly, we observed a significant decline in c-MET mRNA expression in both cell types stimulated with HGF 4Cys-4Ala (Right panel, Supplementary Fig. 9b). Increased c-MET expression might be a crucial determinant for the overall balance of the HGF/c-MET cascade in cells and could be a trigger for the malignant transformation of normal cells. These data suggest that induction of the MMP9 receptor is correlated with overall c-MET expression in an HGF-dependent manner and could be regulated by minimally mutating the four core cysteines in the PAN domain of HGF.

**Fig. 4:**
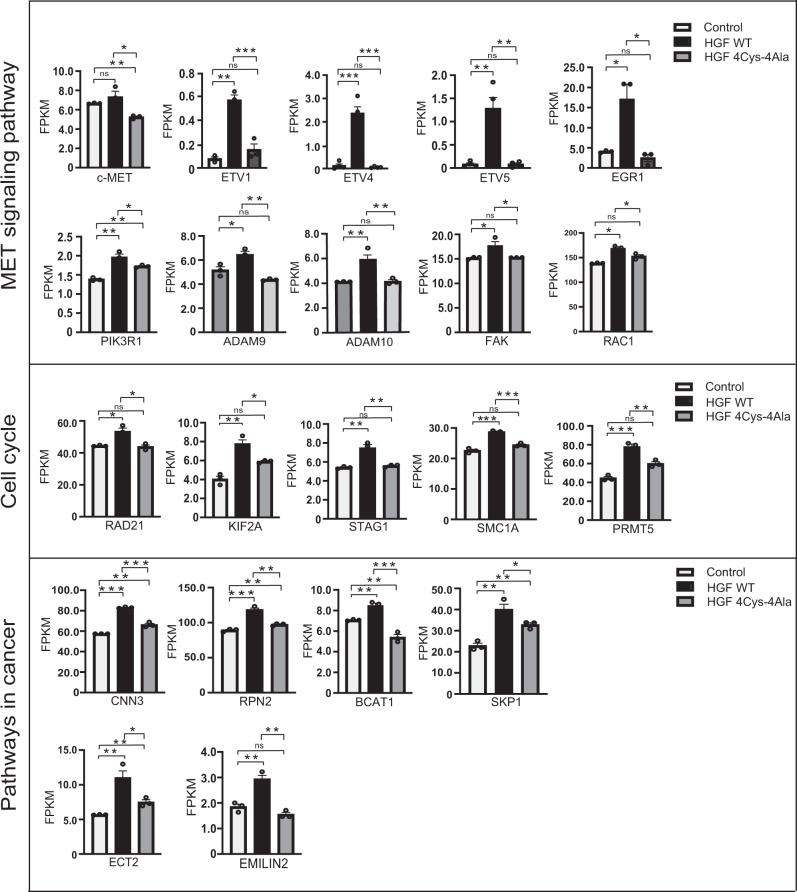
Transcriptome analysis post HGF stimulation in 293T cells. Differential expression analysis by RNA seq in 293T cells following HGF WT and HGF 4Cys-4Ala treatment as indicated confirms that core cysteines in HGF PAN domain are necessary for the expression of a wide range of genes. Responsive genes were normalized to FPKM value for non-treated cells and then normalized to HGF WT treated cells. Data represents the average of three independent biological replicates and *p < 0.05, **p < 0.005 and ***p < 0.0005 were calculated with a student’s t-test.

Based on the apparent PAN domain-dependent transcriptional modulation of MMP9 expression by HGF, we characterized the transcriptional responses of additional downstream genes in the HGF/c-MET signaling pathway. Total RNA was extracted from 293T cells following a 24 h post treatment with wild-type HGF or 4Cys-4Ala HGF. Based on this analysis, we identified significant differences in the expression of genes previously implicated in c-MET signaling, cell cycle regulation, and cancer-related processes (Fig. 4; Supplementary Fig. 10b). Specifically, we observed a striking reduction in the expression levels of the downstream targets of the c-MET signaling cascade, ETS translocation variants 1, 4, and 5 (ETV1, ETV4, ETV5), in cells stimulated with HGF 4Cys-4Ala compared to wild type HGF. These transcription factors are members of the polyoma enhancer activator 3 (PEA3) subgroup of the E–twenty six (ETS) family and confer resistance to early growth factor response (EGFR) targeted therapy in lung cancer^39^. Some known target genes of PEA3 are matrix metalloproteinase-2 (MMP2), matrix metalloproteinase-7 (MMP7) and MMP9, which are well recognized for their role in the invasiveness of cancer^40^. Mutating the core cysteine residues in the PAN domain led to reduced expression of early growth response 1 (EGR1), as well as the metalloproteinases, A disintegrin and metalloprotease domain-containing protein 9 and 10 (ADAM9 and ADAM10), all of which were previously linked to increased adhesion and cancer progression including, hepatocellular carcinoma, and triple negative breast cancer via the AKT/NF-κB axis^41–43^. Hyperactivation of focal adhesion kinase (FAK) and PI3K/receptor for activated C kinase 1 (RAC1) are responsible for metastasis progression and mutations of the core cysteines in the HGF PAN domain also resulted in their reduced expression. Apart from these examples, relative expression of proteins involved in DNA damage repair (double-strand-break repair protein or RAD21), chromosomal integrity and cohesion (structural maintenance of chromosomes protein 1 A or SMC1A), cell cycle progression (kinesin family member 2 A or KIF2A), posttranslational modification, and RNA splicing (protein arginine N-methyltransferase 5 or PRTM5) were also reduced. (Fig. 4)^44–47^. Furthermore, this analysis revealed well-known cancer biomarkers whose expression levels were affected by mutating the core cysteine residues. These biomarkers included calponin-3 or CNN3 (marker for colorectal cancer), dolichyl-diphosphooligosaccharide-protein glycosyltransferase subunit 2 or RPN2 (which inhibits autophagy and upregulates MMP9 expression), branched chain amino acid transaminase 1 or BCAT1 (which promotes hepatocellular carcinoma and chemoresistance), ski interacting protein or SKIP, epithelial cell transforming sequence 2 oncogene or ECT2 (overexpressed in different cancers including non-small cell lung cancer), and elastin microfibril interfacer 2 or EMILIN2 (which promotes angiogenesis and inflammation)^48–53^.

## Discussion

c-MET receptor, which is a receptor tyrosine kinase (RTK) family, plays essential roles(s) in number of cellular processes which includes cell proliferation, survival, morphogenesis, and motility^7^. Abnormal activation of c-MET leads to the progression of tumor growth and metastatic cancer cells, which makes it an important for drug target for cancer treatments^54^. We report here experimental evidence that strongly supports the role of the PAN domain of HGF in providing the functional catalytic core and plays a crucial role in c-MET binding. Although our MD simulation data suggested that there is no change in overall structure of HGF because of PAN mutation, stable cross-linking of c-MET and HGF occurs only in the presence of intact PAN domain in HGF. Additionally, we demonstrated that mutating core cysteine residues in the HGF PAN domain results in a cascade of negative regulation of HGF/c-MET signaling, which starts with attenuated HGF degradation, impaired c-MET interaction, disrupted perinuclear localization, and is followed by a subsequent lack of phosphorylation for c-MET and its downstream targets AKT, ERK and STAT3 and c-MET ubiquitination.

**Fig. 5:**
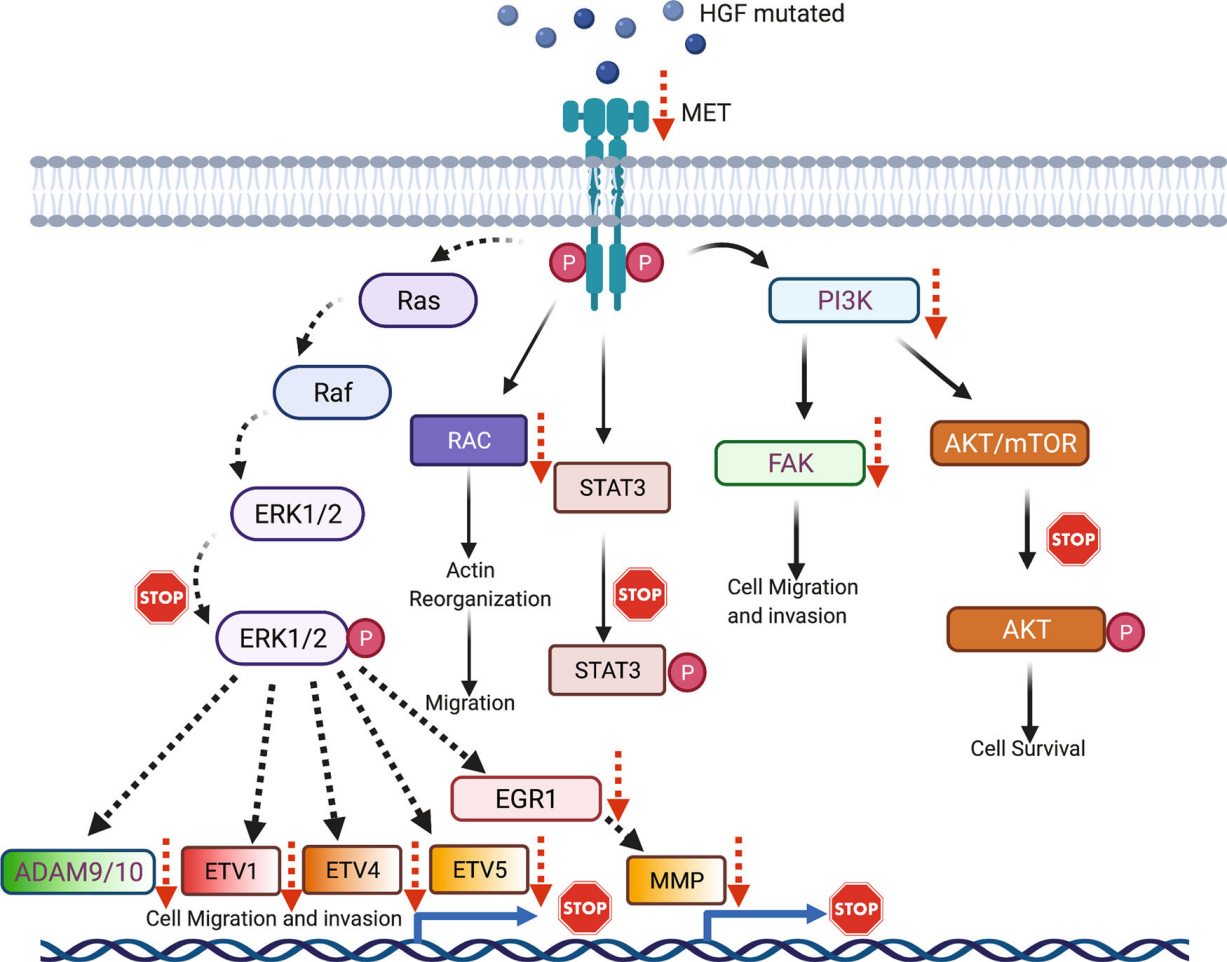
A proposed model showing how conserved cysteines in HGF PAN domain determines the fate of c-MET signaling cascade. PAN domain provides the catalytic core to HGF for c-MET interaction leading towards initiation of the entire downstream HGF/c-MET axis. Alteration in conserved cysteine residues blocks the activation of several transcription factors and effector molecules which otherwise stimulate cell migration, cell invasion, proliferation, and cell motility. Figure created with BioRender.com.

This disruption of upstream events was confirmed by the lack of transcriptional activation of markers genes associated with cell migration and invasion including ADAM9/10, EGR1, ETV1, ETV4, ETVE5, and MMP9 (Fig. S10B). Targeting the PAN domain in HGF could act as a multi-pathway target as it entirely shuts down the c-MET signaling cascade and downstream expression of relevant proteins known for their role in cancer prognosis and other diseases (Fig. 5). Taken together, the PAN domain and its core cysteine residues are essential for HGF/c-MET signaling in human cells. The observed enhanced stability of HGF with mutated cysteine residues is due to the lack of its stable interaction with c-MET. Considering the results described in this study, it would be essential to determine the molecular mechanism(s) by which the PAN domain regulates the c-MET interaction, which requires additional studies. This report identifies the HGF PAN domain as a potential site for c-MET binding and MET signaling cascade activation. Targeting the PAN domain can block cell proliferation by inhibiting MAPK phosphorylation and altering the expression of different cancer biomarkers. These observations point to the exciting prospect of treating HGF-overexpressing tumors by selectively targeting the PAN domain.

## Materials and methods

### PAN domain sequence alignment

Proteins with PAN domains were identified from Uniprot and were selected from 14 model organisms according to Bateman et al.^55^. The PAN domain coordinates from UniProt were used to extract the corresponding sequences from the full-length proteins, which were then aligned with MAFFT linsi^56^. The alignment was visualized with Geneious^41^ and a phylogenetic tree accompanying the alignment was constructed via the neighbor-joining method with Geneious (Supplementary Data 3).

### GO enrichment of 14 PAN domain categories

GO terms of each category were extracted from InterPro2GO database from InterPro^57^. GO enrichment analysis for each category against all PAN domain genes was performed by Fisher’s exact test via the TopGO package^58^. Only the biological process GO category was used for GO enrichment.

### Mammalian cell culture, transfection, and drug treatment

HeLa, HEK 293T, and Glioblastoma U87 cells were obtained from ATCC and maintained in a humidified atmosphere at 5% CO_2_ in Dulbecco’s Modified Eagle’s (DMEM) complete medium (Corning) supplemented with 10% fetal bovine serum (FBS; Seradigm) in 37 °C. Plasmid transfections were done with TransIT-LT1 (Mirus Bio) per the manufacturer’s instructions.

### Plasmids and recombinant proteins

Flag-HGF (GeneBankTM accession number NM_000601.5) and various mutants of Flag-HGF, cloned into pcDNA 3.1 were obtained from GenScript. Purified Flag tagged HGF wild type, HGF C70A, HGF C74A, HGF C84A, HGF C96A, and HGF 4Cys-4Ala proteins were customized and synthesized using HD CHO-S cell expression (GenScript) (Supplementary Fig. 11). Briefly, Target DNA sequence was designed, optimized and synthesized, and sub-cloned into pcDNA3.4 vector and transfected into CHO-S cells. Following expression, Cell culture broth was centrifuged and followed by filtration. Filtered cell culture supernatant was loaded onto an affinity purification column (Anti-DYKDDDK G1 Affinity Resin 3 ml) at an appropriate flowrate (1 ml/min). After washing and elution with appropriate buffers (Washing buffer: PBS, pH7.2; Elution buffer: 0.1 M Gly-HCl, pH 3.5), the eluted fractions were pooled, and buffer exchanged to the final formulation buffer (PBS, pH7.2). The purified protein was analyzed by SDS-PAGE, Western-blot, HPLC analysis to determine the molecular weight and purity. c-MET-C-GFPSpark® Clone was obtained from SinoBiological. Purified His tagged c-MET protein was obtained from R&D Systems (#358-MT-100/CF).

### Immunofluorescence and confocal microscopy

HeLa cells were seeded on coverslips in 24 well plates. Where indicated, cells were transfected with Flag-HGF WT and different PAN domain mutants for 36 h, followed by fixation in 4% paraformaldehyde. Next, the cells were permeabilized with 0.5% Triton X-100 in PBS, washed, and then blocked for 30 min at room temperature with 5% BSA in PBS. Cells were incubated with primary antibodies in 5% BSA in 1X-PBS with 0.5% Triton X-100 for 1 h at room temperature. After washing the cells were incubated with appropriate secondary antibodies in 5% BSA in PBST for 30 min at room temperature. DNA was counterstained with 1 µg/mL Hoechst 33342 and mounted with Fluorimount G (Southern Biotech). Cells were imaged using a Zeiss LSM 710 confocal microscope. For c-MET and STAT3 localization assays, cells were serum-starved for 24 h before the stimulations. Cells were then stimulated with 100 ng mL^−1^ of WT HGF and or 4Cys-4Ala HGF purified proteins for the indicated amount of time at 37 °C and treated as described above.

### Antibodies

The following commercial antibodies and the indicated concentrations were used in this study. HA antibody (HA.C5 #18181; 1:1000) was purchased from Abcam. Flag (#2368S; 1:1000), (Met (clone 25H2 #3127; 1:1000), Phospho-Met (Tyr 1234/1235) (clone D26 # 3077; 1:1000), Stat3 (clone 124H6 #9139; 1:1000), Phospho-Stat3 (Tyr 705) (clone D3H7 #9145; 1:1000), Akt (pan) (clone 40D4 #2920; 1:1000), Phospho Akt (Thr 308) (clone 244F9 #4056; 1:1000), p44/42 MAPK (Erk1/2) (#9102; 1:1000), Ubiquitin (clone E4I2J) (#43124; 1:1000), Met (clone D1C2) (#8198; 1:1000) and Phospho-p44/42 MAPK (Erk1/2) (Thr202/Tyr204) (clone D13.14.4E) (#4370; 1:1000) were purchased from Cell signaling. M2 anti Flag Mouse antibody (#SLBT7654; 1:5000) and Actin (#087M4850; 1:10,000) were purchased from Sigma. HA (#902302; 1:1000) antibody was purchased from Biolegend. Invitrogen GFP (clone A-11122) (1:1000) was purchased from Thermo Fisher Scientific. Met Antibody (clone D-4) (#sc-514148; 1:500) was purchased from Santa Cruz Biotechnology. Streptavidin-Horseradish Peroxidase (HRP) Conjugate was obtained from Thermo Scientific (#33073). Chemiluminescence detection was performed according to the manufacturer’s instructions (Amersham ECL Western Blotting Detection Reagent kit) followed by exposure using Chemidoc gel-documentation system (BioRad). For imaging using Li-Cor, secondary antibodies for western blotting were purchased from LI-COR Biosciences.

### Western blotting and immunoprecipitation

For in vivo stimulation experiments, cells were grown for 36 h and then stimulated with HGF WT and HGF 4Cys-4Ala where indicated (100 ng mL^−1^), washed with PBS, and lysed. Briefly, cell extracts were generated on ice in EBC buffer, 50 mM Tris (pH 8.0), 120 mM NaCl, 0.5% NP40, 1 mM DTT, and protease and phosphatase inhibitors tablets (Thermo Fisher Scientific). Extracted proteins were quantified using the PierceTM BCA Protein assay kit (Thermo Fisher). Proteins were separated by SDS acrylamide gel electrophoresis and transferred to IMMOBILON-FL 26 PVDF membrane (Millipore) probed with the indicated antibodies and visualized either by chemiluminescence (according to the manufacturer’s instructions) or using a LiCor Odyssey infrared imaging system.

For immunoprecipitation, endogenous c-MET was immunoprecipitated on c-MET antibody-bound beads (Dynabeads Protein G from Thermo Fisher) and Flag-tagged HGF WT and HGF 4Cys-4Ala were in-vitro translated (T_N_T quick coupled Transcription/Translation system, Promega) and were incubated with the bead bound c-MET for 4 h at 4 °C. Beads were then washed and proteins resolved by SDS-PAGE and analyzed by western blotting as above.

### Cross-linking assay

The hetero tri-functional cross-linker Sulfo-SBED (2-[6-(biotinamido)-2-(p-azidobenzamido)-hexanoamido]ethyl-1,39-dithiopropionate from Thermo Scientific # 33073) was used for studying the interaction between HGF and c-MET per the manufacturer’s instructions. Briefly, purified Flag-HGF WT or Flag-HGF 4Cys-4Ala (1 μg each) was used as a bait protein and labeled with Sulfo-SBED for 30 min at room temperature in the dark. Unincorporated cross-linker was removed by dialyzing the reaction mixture against 1X Label Transfer Buffer at 4 °C for overnight in the dark. SBED-labeled HGFs were added to the purified His-c-MET protein and incubate for 45 min. Following UV cross-linking for 15 min (6-watt hand-held lamps at distance of 5 cm), the disulfide bond of Sulfo-SBED was cleaved by 2-mercaptoethanol resulting in a biotin label attached to the interacting c-MET protein conjugate. Samples were divided into two equal parts and the biotin labeling of c-MET was analyzed by electrophoresis, followed by western blotting using Streptavidin-HRP as probe for one part and anti-His and anti-Flag antibodies for the other part. For the in-vivo cross-linking, HEK293T lysate expressing GFP-C-MET protein was substituted for purified His-c-MET and incubated at 4 °C for 45 min in the dark. After UV cross-linking (6-watt hand-held lamps at distance of 5 cm), biotin-containing complexes were immunoprecipitated with streptavidin beads (1 h at 4 °C, Pierce™ Streptavidin Magnetic Beads). Immunoprecipitates were washed three times with PBS and analyzed by Western blotting on reducing (mercaptoethanol-containing) SDS-gels using anti-GFP, anti-Flag antibodies, and Streptavidin-HRP as a probe.

### In vivo ubiquitination

293T cells were transfected with the construct encoding c-MET-C-GFPSpark. Cells were stimulated with either HGF WT or HGF 4Cys-4Ala as indicated following serum starvation. Cells were collected at indicated time points and washed with ice-cold PBS, lysed in ice-cold buffer containing 10 mM Tris-HCl (pH 8), 150 mM NaCl, 0.1% SDS, 20 mM NEM (*N*-ethylmaleimide) supplemented with protease and phosphatase inhibitors tablets (Thermo Fisher Scientific). The lysates were cleared by centrifugation at 10,000 × *g* at 4 °C for 20 min, followed by preclearance using protein G-beads (protein G from Thermo Fisher). 500 µg of each precleared lysate was incubated with anti-MET antibody and protein-G beads for overnight at 4 °C with rotation. The samples were then washed three times with the lysis buffer and eluted with SDS-gel loading buffer (with reducing agent added). Proteins were resolved by SDS-PAGE and immunoblotted with the indicated antibodies.

### Cell proliferation assay

3-(4, 5-dimethylthiazol-2-yl)-2, 5-diphenyl tetrazolium bromide (MTT) assay was used to determine cell viability. HEK293T and U-87-MG cells were plated in 96-well plates with 500 cells per well in triplicate in serum-free medium for 24 h prior to HGF stimulations. 100 ng mL^−1^ HGF WT, HGF 4Cys-4Ala, or both were added to the cells and incubated for 24 h prior to the addition of MTT solution (Abcam, Inc # ab211091) and cell viability was measured according to the manufacturer’s instruction. The assays were performed in triplicate and the experiment was repeated three times. Data were expressed as the mean ± SD. Statistical analyses were performed. *P < 0.05 was considered to indicate a statistically significant difference.

### Quantitative real-time PCR and RNA-Seq analysis

Total RNA was extracted from glioblastoma U-87 MG and HEK293T cell line 24 h post HGF stimulation using TRIzol reagent (Invitrogen), and reverse transcription was performed using the SuperScript II RT kit (Integrative DNA Technologies) with total RNA (1 μg) according to the manufacturer’s instructions. The c-MET and MMP9 mRNA expression levels were detected by conventional RT-PCR with Taq DNA Polymerase, Recombinant (Invitrogen, no. 10342-020). Glyceraldehyde-3-phosphate dehydrogenase (GAPDH) was used as the internal control. The specific primers for c-MET, MMP9 and GAPDH were designed with Primer Premier software. The primers used were c-MET, forward: 5′-TTAAAGGAGACCTCACCATGTAATC-3′ and reverse: 5′-CCTGATCGAGAAACCACAACCT -3′; MMP9, forward: 5′-GATCCAAAACTACTCGGAAGACTTG-3′ and reverse: 5′-GAAGGCGCGGGCAAA-3′ and GAPDH, forward: 5′-TTGCCATCAATGACCCCTTCA-3′ and reverse: 5′-CGCCCCACTTGATTTTGGA-3′. The PCR reaction was performed according to the manufacturer’s instructions. The PCR conditions were as follows: amplification reaction protocol was performed for 35 cycles consisting of 30 s at 94 °C (denaturation), annealing 30 s at 45 °C and extension 30 s at 72 °C.

For RNA-seq analysis, Total RNA was extracted from the HEK293T cell line 24 h post HGF stimulation using TRIzol reagent (Invitrogen) according to the manufacturer’s instructions. Quantification and quality control of isolated RNA was performed by measuring absorbance at 260 and 280 nm on a NANODROP ONEC spectrophotometer (Thermo Scientific, USA). The RNA-seq run was performed with four biological replicates. Library prep and sequencing was performed by BGI using the DNBSEQ-G400 platform which generated 100 bp paired-end reads. The raw RNA-seq reads have been deposited at NCBI under BioProject ID PRJNA718097. And the link to access the data is. Clean reads were aligned to the human reference genome GRCh38. Reads were mapped with bowtie2 v2.2.5^59^. Expression levels for RNAs were calculated using fragments per kilobase per million reads (FPKM) values with RSEM v1.2.8^60^. Differential expression analysis was performed with DESeq2 and genes with an adjusted *p*-value less than 0.05 were considered differentially expressed^61^.

### Molecular dynamics simulations

MD simulations were initiated from the top five models generated with ColabFold^27,62^ of the WT and 4Cys-4Ala mutant PAN domain. The program *tleap* from AmberTools20^63^ was used to prepare the parameter and coordinate files for each structure. The ff14SB force field^64^ and TIP3P water model^65^ were used to describe the protein and solvent, respectively. Energy minimization was performed using *sander* from AmberTools20. At least a 12 Å solvent buffer between the protein and the periodic images. Sodium and chloride ions were added to neutralize charge and maintain a 0.10 M ion concentration. The simulations were performed with OpenMM version 7.5.1^66^) on the Cuda platform (version 11.0.3) using Python 3.8.0. ParmEd was used to incorporate the force field parameters into the OpenMM platform^67^. The Langevin integrator and Monte Carlo barostat were used to maintain the systems at 300 K and 1 bar, respectively. Direct non-bonded interactions were calculated up to a 12 Å distance cutoff. All bonds involving hydrogen atom were constrained to their equilibrium values. The particle mesh Ewald method was used to compute long-range Coulombic interactions^68^. A 2 fs integration time step was used with energies and positions written every 2 ps.

### Simulation analysis

Analyses of MD trajectories were performed using Python 3.8.0 and the MDAnalysis version 1.0.1^69,70^. Matplotlib was used to plot the data.

### Statistics and reproducibility

Statistical analyses were performed on individual experiments, as indicated, with GraphPad Prism 8 Software using an unpaired t-test, equal variance, for comparisons between two groups. A *P*-value of *P < 0.05 was considered statistically significant.

### Reporting summary

Further information on research design is available in the Nature Research Reporting Summary linked to this article.

## Supplementary information

Supplementary Information

Description of Additional Supplementary Files

Supplementary Data 1

Supplementary Data 2

Supplementary Data 3

Reporting Summary

## Data Availability

This manuscript has been authored by UT-Battelle, LLC under Contract No. DE-AC05-00OR22725 with the U.S. Department of Energy. The United States Government retains and the publisher, by accepting the article for publication, acknowledges that the United States Government retains a non-exclusive, paid-up, irrevocable, world-wide license to publish or reproduce the published form of this manuscript, or allow others to do so, for United States Government purposes. The Department of Energy will provide public access to these results of federally sponsored research in accordance with the DOE Public Access Plan (http://energy.gov/downloads/doe-public-access-plan). All data are available in the main text or the Supplementary Information and the Supplementary Data 1–3. Uncropped gel scans for all presented western blots are included in the Supplementary Figs. 12–16. The raw RNA-seq reads have been deposited at NCBI under BioProject ID PRJNA718097 and the link to access the data is.
