# Supplementary material for: Core cysteine residues in the Plasminogen-Apple-Nematode (PAN) domain are critical for HGF/c-MET signaling
Source: Commun Biol. 2022 Jul 1;5:646. doi: 10.1038/s42003-022-03582-8 (PMC9249922; doi:10.1038/s42003-022-03582-8)
Supplement: Supplementary file 1 — Supplementary Information [file 42003_2022_3582_MOESM1_ESM.pdf]

Supplementary Materials for

Core cysteine residues in the Plasminogen-Apple-Nematode (PAN) domain are critical for HGF/c-MET signaling

Debjani Pal[1†], Kuntal De[1†], Carly M. Shanks[1], Kai Feng[1], Timothy B. Yates[1,2], Jennifer Morrell-Falvey[1], Russell B. Davidson[1], Jerry M. Parks[1], Wellington Muchero[1,2*].

Correspondence to: mucherow@ornl.gov

This PDF file includes:

Legends for Supplementary Figure(s). 1 to 16

Supplementary Figure(s). 1 to 16

Supplementary Figure 1. The HGF PAN domain is comprised of a core of four conserved cysteine residues. (a) Multiple alignment of the sequences of PAN domain of representative proteins from different organisms highlights the position of four conserved cysteines. (b) Schematic diagram of amino acid sequence represents HGF PAN domain along with the four marked conserved cysteines (Cys70, Cys74, Cys84 and Cys96) and the subsequent mutant version where conserved cysteines were mutated to alanine (Ala70, Ala74, Ala84 and Ala96).

Computational secondary structure prediction (Chimera) for HGF PAN domain identifies the residues forming β-strands and α-helix in PAN domain.

Supplementary Figure 2. The 3D conformation of four reported mutation sites located in the PAN domain of HGF.

Supplementary Figure 3. Root-mean-square deviation (RMSD) time evolution plots from WT and 4Cys-4Ala MD simulations. RMSD profiles and histograms for (a) Wild-type and (b) 4Cys-4Ala mutant. For both datasets, the reference structure was the initial coordinates of the top WT model. RMSDs were calculated for all heavy atoms after alignment of all Ca atoms.

Supplementary Figure 4. Representative structures of the WT and 4Cys-4Ala PAN domain from MD simulations. (Left) Ca alignment of both structures to the initial coordinates of the top WT model. (Right) Close-up of the mutation positions for the two systems. In the absence of the disulfide pairs, the 4Cys-4Ala mutant maintained near equivalent positioning of the mutated residues.

Supplementary Figure 5. The PAN domain of HGF is necessary for c-MET activation. (a and b). HeLa cells were stimulated with HGF WT and HGF 4Cys-4Ala and expression of the proteins

related to c-MET signaling were examined by immunoblotting at the indicated times. Representative blot images from n=2 experiments.

Supplementary Figure 6. Mutations of individual cysteines in the PAN domain abrogate downstream c-MET signaling. 293T cells were stimulated with purified HGF WT, HGF C70A, HGF C74A, HGF C84A and HGF C96A mutants and phosphorylation of c-MET was examined by immunoblotting at the indicated times. Representative blot images from n=2 experiments.

Supplementary Figure 7. Transfer of the biotin-tag from HGF into c-MET. (a) *in vitro* cross-linking of purified HGF and purified c-MET. Purified 1 ug of Flag-HGF WT or Flag-HGF 4Cys-4Ala was activated by incubating with a biotin-containing trifunctional cross-linking reagent (Sulfo-SBED from Thermo). Activated HGF was then incubated with purified His-c-MET. After UV cross-linking (15 min), Sulfo-SBED biotin label transfer to His-c-MET from Flag-HGF WT or 4Cys-4Ala were analyzed by western blotting on reducing SDS-gels using anti-His, anti-Flag, and Streptavidin-HRP as a probe. Representative image of n=3 biological replicates. (b) Quantification of His-c-MET biotinylation in (a). Streptavidin band intensities for c-MET (a, top panel) were normalized to the streptavidin band intensities for labeled HGF protein (a, middle panel) before being cross-linked c-MET. Data are represented as mean ± SEM, *** $p < 0.0005$ (Student's t test) and $n = 3$ biological replicates. (c) Purified biotinylated HGF 4Cys- 4Ala could not cross-link with GFP-tagged c-MET expressed in 293T cells. The cross-linked complex was

immunoprecipitated on Streptavidin beads, and the amount of bound GFP-c-MET was confirmed following western blot using anti-GFP as a probe.

Supplementary Figure 8. STAT3 phosphorylation is abrogated following HGF PAN mutation. HeLa cells were treated with HGF WT and HGF 4Cys-4Ala for the indicated times, and immunoblotting was performed using anti-pSTAT3. Total STAT3 and actin were used as a loading control.

Supplementary Figure 9. HGF stimulates cell proliferation and MMP9 expression in PAN domain dependent manner. (a) Mutations on the conserved cysteines in HGF PAN domain downregulate cell proliferation. 293T and U-87 MG cells were plated in 96-well plates in serum-free medium for 24 hours. HGF WT and HGF 4Cys-4Ala were added to the cells where indicated and plates were incubated for 24 h. MTT reagents were added, and the absorbance was read at 492 nm. A significant increase in the proliferation for both cells were observed when treated with HGF WT (*$p<0.05$). (b) Quantitative PCR analysis for MMP9 and MET mRNA expression. 293T and U-87 MG cells were serum starved before HGF treatment. Treatment was given for 24 hours and MMP9 and MET mRNA expressions were detected by conventional RT PCR. The graph represents the relative mRNA expression normalized to GAPDH control. Data (a and b) are represented as mean ± SD, n=3 independent biological replicates and *$p<0.05$ (Student's t test).

Supplementary Figure 10. (a) MTT cell proliferation assay. 293T cells were transfected with Flag-HGF WT and Flag-HGF 4Cys-4Ala. 24 hr post transfection cells were splitted and re-plated in 96-well plates in serum-free medium for 24 hours. Plates were incubated for the indicated time. MTT reagents were added, and the absorbance was read at 492 nm. A significant increase in the proliferation for cells was observed when transfected with HGF WT (* $p<0.05$). (b) Heatmap of selected genes. Heat maps displaying pattern of expression for the candidates involved in Met signaling, cell cycle and invasion. Genes are depicted based on their expression ratios across three RNA seq comparison. Colors range from bright green (upregulation; log_2 ratio over control) to bright red (downregulation; log_2 ratio over control).

Supplementary Figure 11. Certificate of analysis of Flag tagged HGF WT, HGF C70A, HGF C74A, HGF C84A, HGF C96A and HGF 4Cys- 4Ala proteins using SDS PAGE and Western blot.

Supplementary Figure 12. Uncropped gel scans for all presented Western blots. (a) corresponds to Fig. 1a; (b) corresponds to Fig. 1c; (c) corresponds to Fig. 1d; (d-e) corresponds to Fig. 2a.

Supplementary Figure 13. Uncropped gel scans for all presented Western blots. (a) corresponds to Fig. 2b; (b) corresponds to Fig. 2e; (c) corresponds to Fig. 3a.

Supplementary Figure 14. Uncropped gel scans for all presented Western blots. (a) corresponds to Supplementary Figure 5a; (b) corresponds to Supplementary Figure 5b; (c) corresponds to Supplementary Figure 6.

Supplementary Figure 15. Uncropped gel scans for all presented Western blots. (a) corresponds to Supplementary Figure 7a; (b) corresponds to Supplementary Figure 7c.

Supplementary Figure 16. Uncropped gel scans for all presented Western blots. Figure corresponds to Supplementary Figure 8.

a

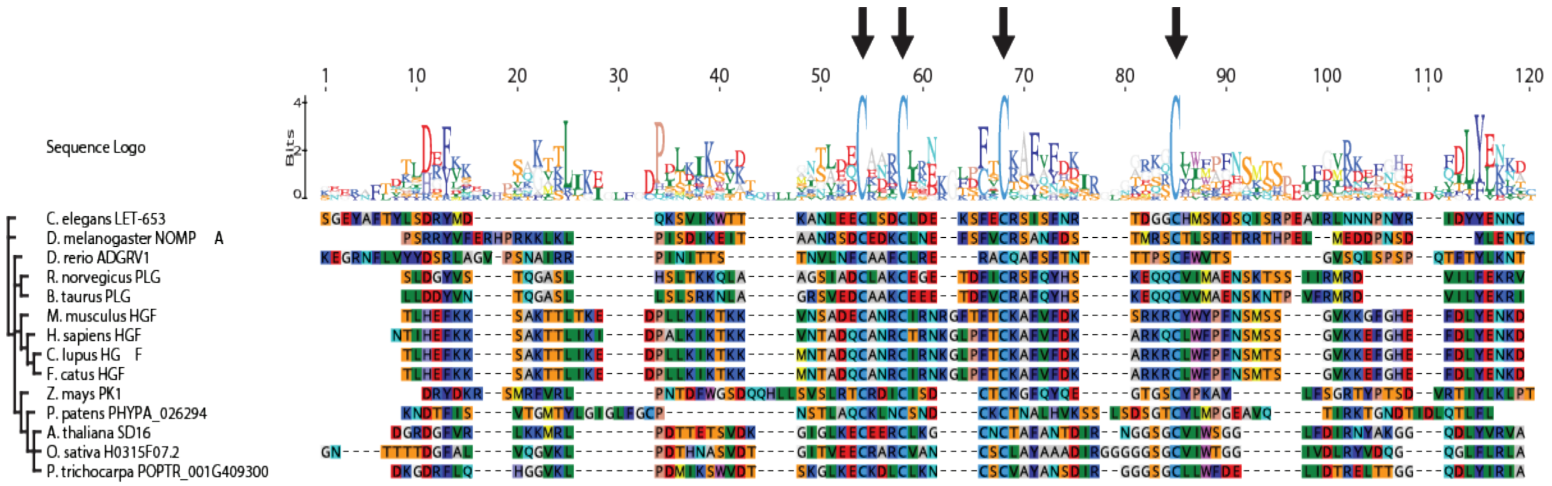
Sequence Logo
Bits
C. elegans LET-653
D. melanogaster NOMPA
D. rerio ADGRV1
R. norvegicus PLG
B. taurus PLG
M. musculus HGF
H. sapiens HGF
C. lupus HGF
F. catus HGF
Z. mays PK1
P. patens PHYPA_026294
A. thaliana SD16
O. sativa H0315F07.2
P. trichocarpa POPTR_001G409300

b

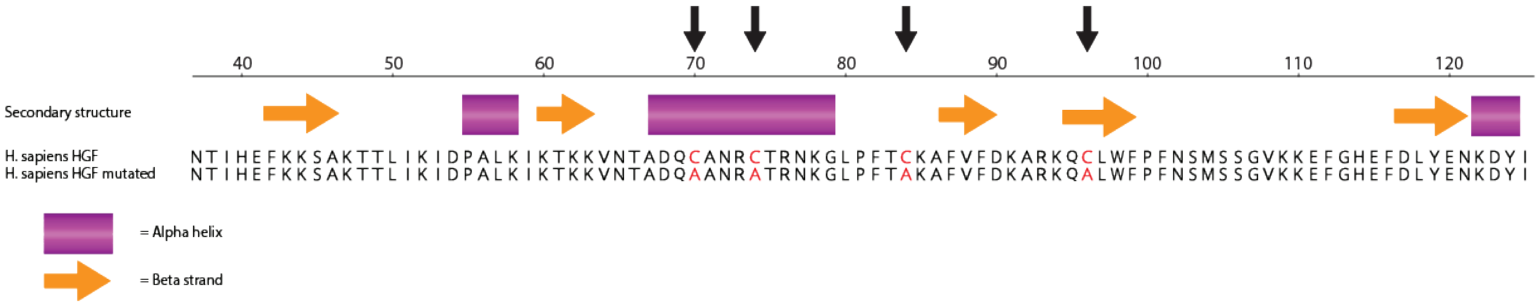
Secondary structure
H. sapiens HGF
NTIHEFKKSAKTTLIKIDPALKIKTKKVNTADQCANRCTRNKGLPFTCKAFVFDKARKQCLWFPFNSMSSGVKKEFGHEFDLYENKDYI
H. sapiens HGF mutated
NTIHEFKKSAKTTLIKIDPALKIKTKKVNTADQAANRATRNKGLPFTAKAFVFDKARKQALWFPFNSMSSGVKKEFGHEFDLYENKDYI
= Alpha helix
= Beta strand

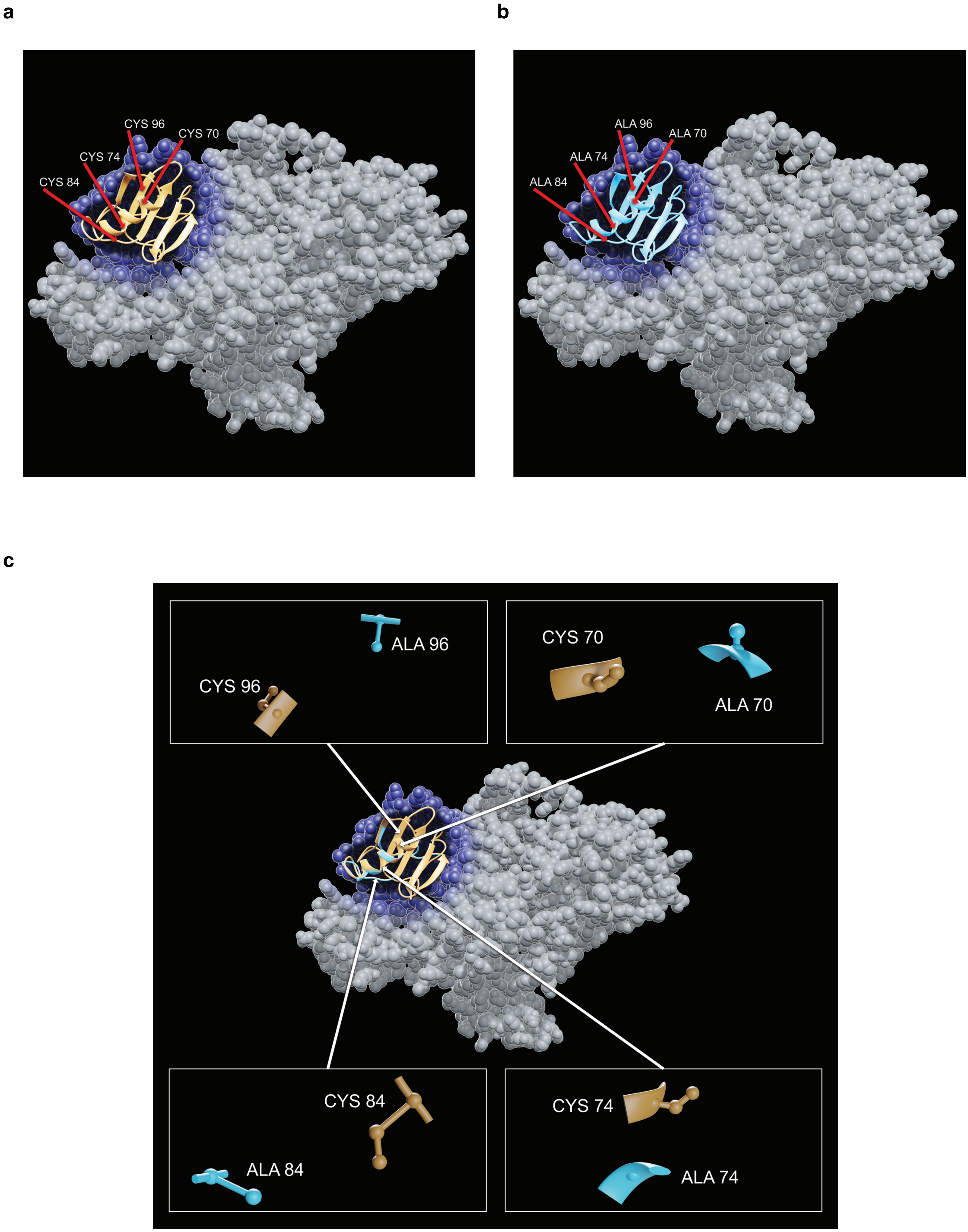

a
CYS 96
CYS 70
CYS 74
CYS 84
b
ALA 96
ALA 70
ALA 74
ALA 84
c
ALA 96
CYS 96
CYS 70
ALA 70
CYS 84
ALA 84
CYS 74
ALA 74

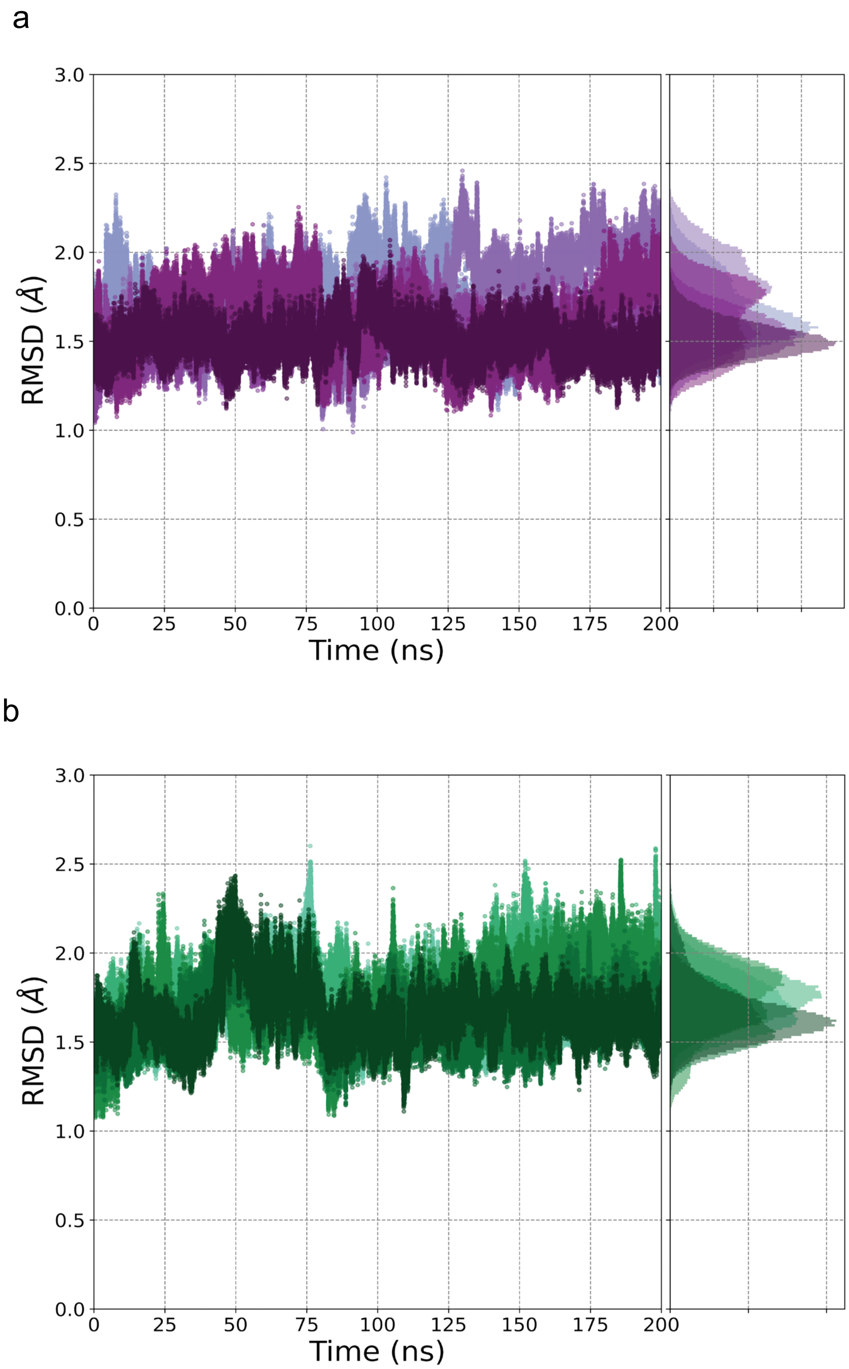
a
b
RMSD (Å)
Time (ns)
0.0
0.5
1.0
1.5
2.0
2.5
3.0
0
25
50
75
100
125
150
175
200

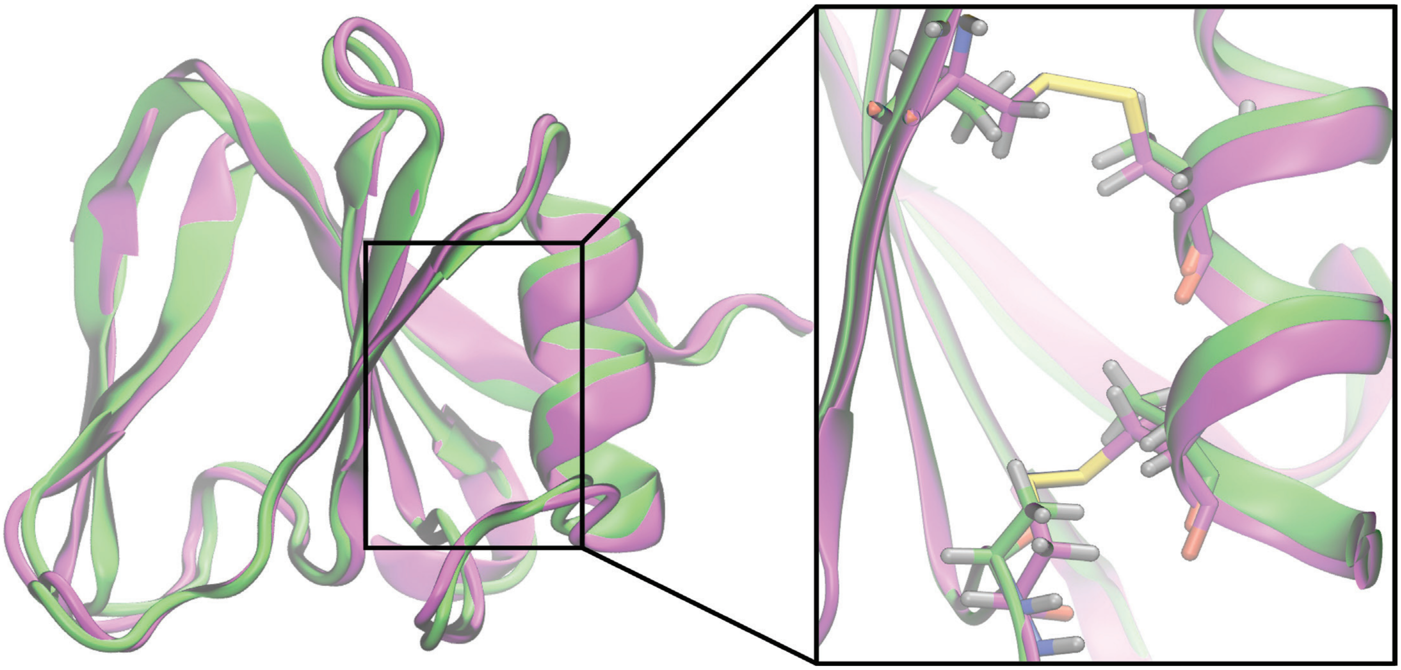

a

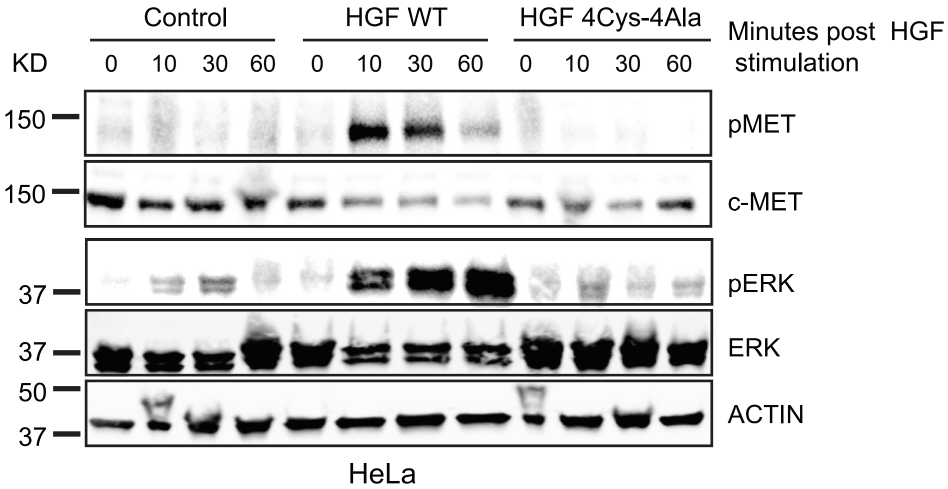
Control
HGF WT
HGF 4Cys-4Ala
Minutes post HGF stimulation
KD
0 10 30 60 0 10 30 60 0 10 30 60
150
150
37
37
50
37
pMET
c-MET
pERK
ERK
ACTIN
HeLa

b

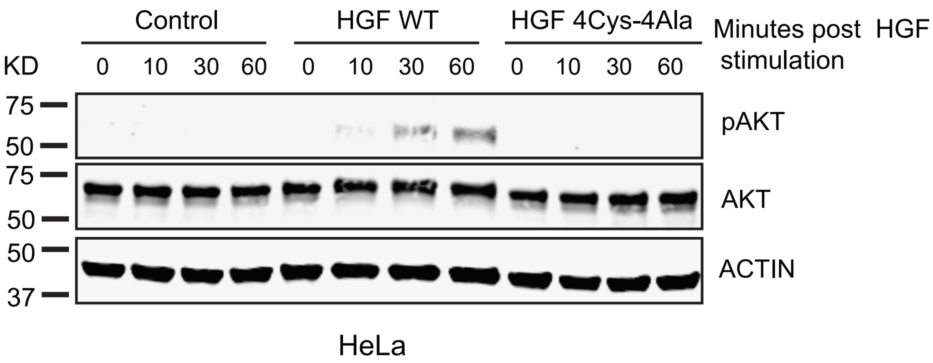
Control
HGF WT
HGF 4Cys-4Ala
Minutes post HGF stimulation
KD
0 10 30 60 0 10 30 60 0 10 30 60
75
50
75
50
50
37
pAKT
AKT
ACTIN
HeLa

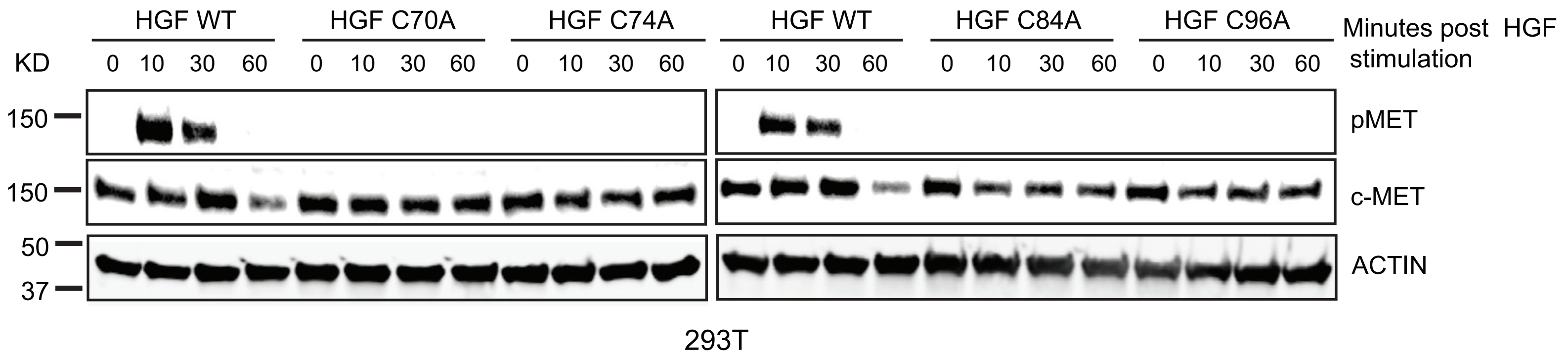
HGF WT
HGF C70A
HGF C74A
HGF WT
HGF C84A
HGF C96A
Minutes post HGF stimulation
KD
0 10 30 60 0 10 30 60 0 10 30 60 0 10 30 60 0 10 30 60 0 10 30 60
150
150
50
37
pMET
c-MET
ACTIN
293T

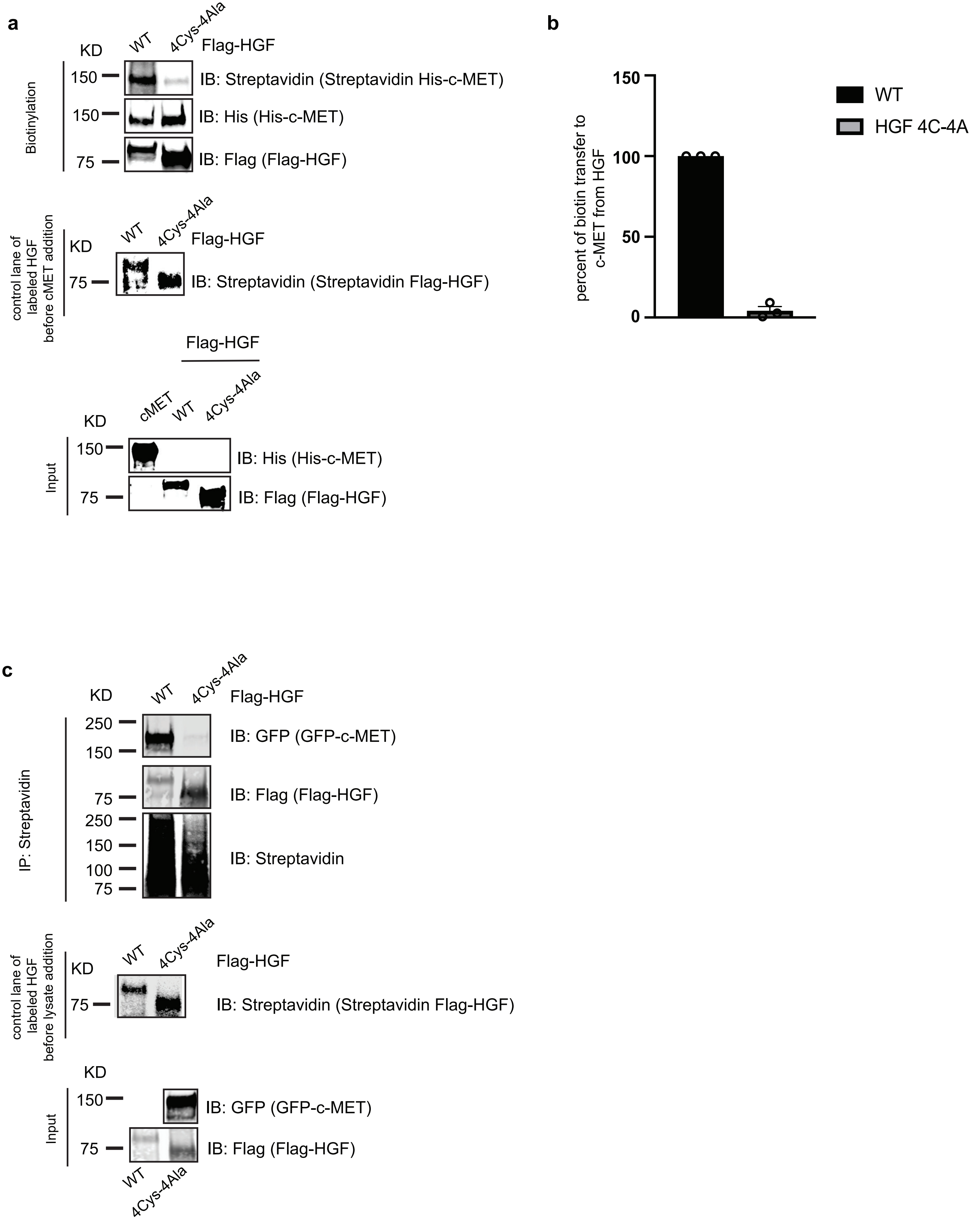

a
KD
WT
4Cys-4Ala
Flag-HGF
Biotinylation
150
IB: Streptavidin (Streptavidin His-c-MET)
150
IB: His (His-c-MET)
75
IB: Flag (Flag-HGF)
control lane of labeled HGF before cMET addition
KD
WT
4Cys-4Ala
Flag-HGF
75
IB: Streptavidin (Streptavidin Flag-HGF)
Flag-HGF
KD
cMET
WT
4Cys-4Ala
Input
150
IB: His (His-c-MET)
75
IB: Flag (Flag-HGF)
b
percent of biotin transfer to c-MET from HGF
150
100
50
0
WT
HGF 4C-4A
c
KD
WT
4Cys-4Ala
Flag-HGF
IP: Streptavidin
250
150
IB: GFP (GFP-c-MET)
75
IB: Flag (Flag-HGF)
250
150
100
75
IB: Streptavidin
control lane of labeled HGF before lysate addition
KD
WT
4Cys-4Ala
Flag-HGF
75
IB: Streptavidin (Streptavidin Flag-HGF)
KD
Input
150
IB: GFP (GFP-c-MET)
75
IB: Flag (Flag-HGF)
WT
4Cys-4Ala

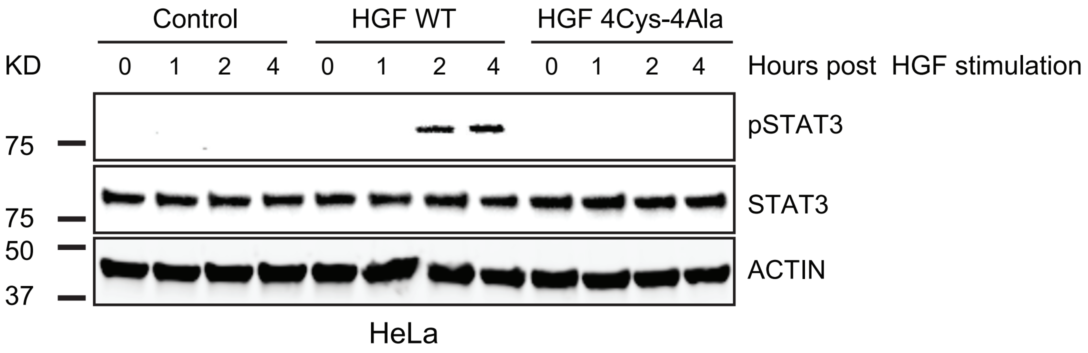
Control
HGF WT
HGF 4Cys-4Ala
KD
0 1 2 4 0 1 2 4 0 1 2 4
Hours post HGF stimulation
75
pSTAT3
STAT3
75
50
ACTIN
37
HeLa

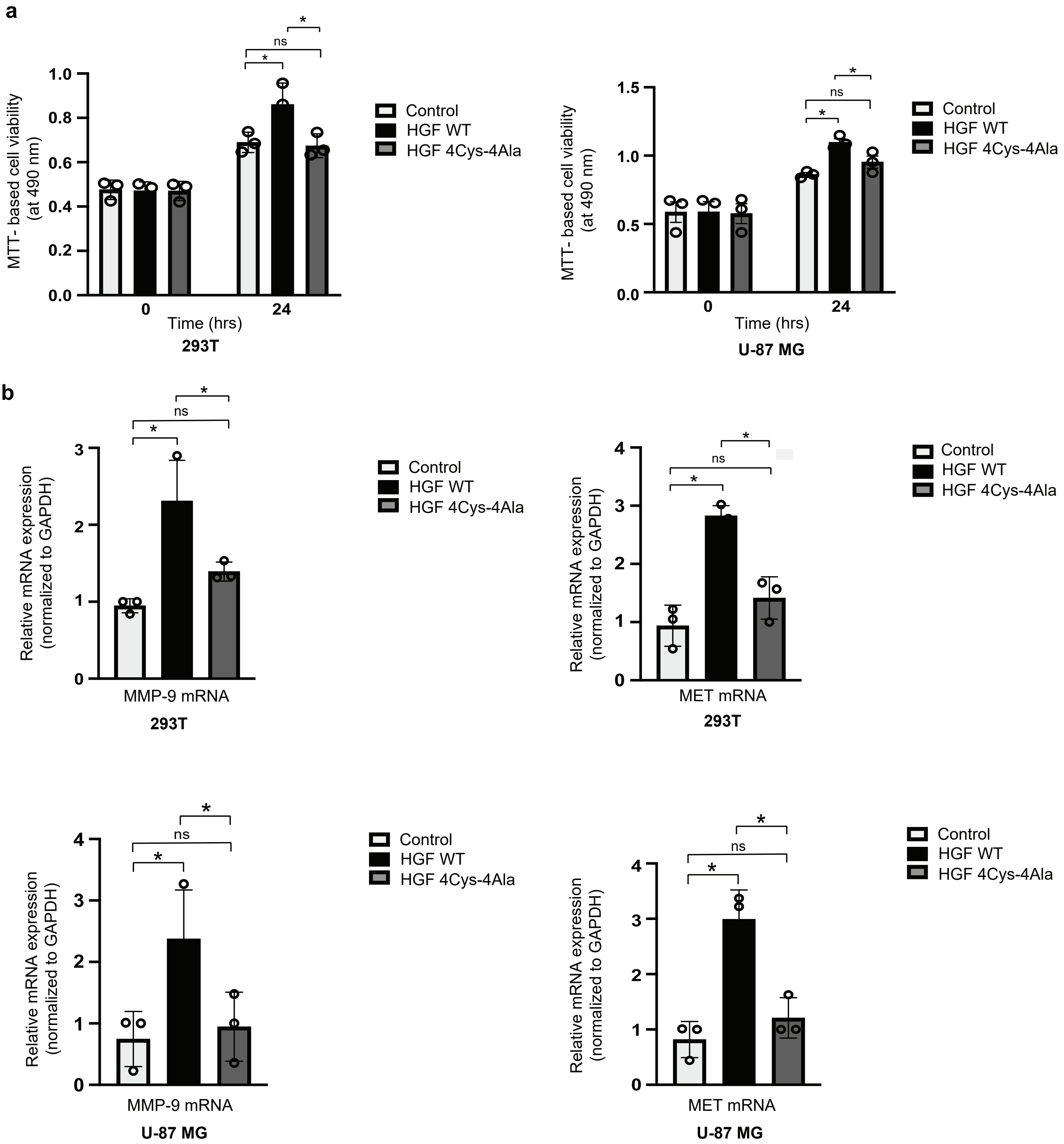
a
MTT- based cell viability (at 490 nm)
1.0
0.8
0.6
0.4
0.2
0.0
ns
*
0
24
Time (hrs)
293T
Control
HGF WT
HGF 4Cys-4Ala
1.5
1.0
0.5
0.0
U-87 MG
b
Relative mRNA expression (normalized to GAPDH)
3
2
1
0
MMP-9 mRNA
4
MET mRNA
U-87 MG

a

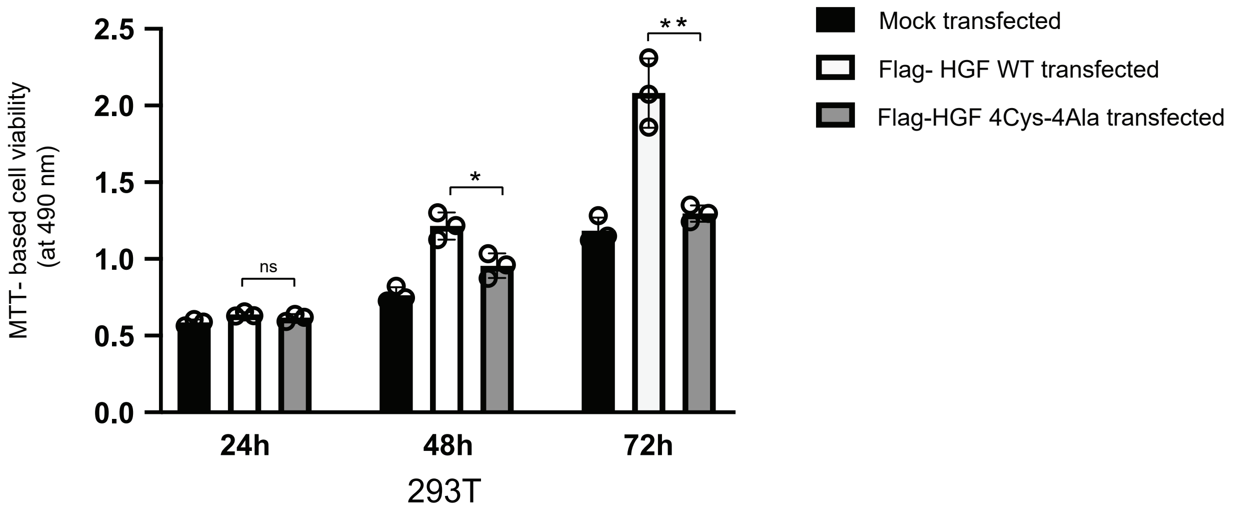

MTT- based cell viability
(at 490 nm)
2.5
2.0
1.5
1.0
0.5
0.0
ns
*
**
24h
48h
72h
293T
Mock transfected
Flag- HGF WT transfected
Flag-HGF 4Cys-4Ala transfected

b

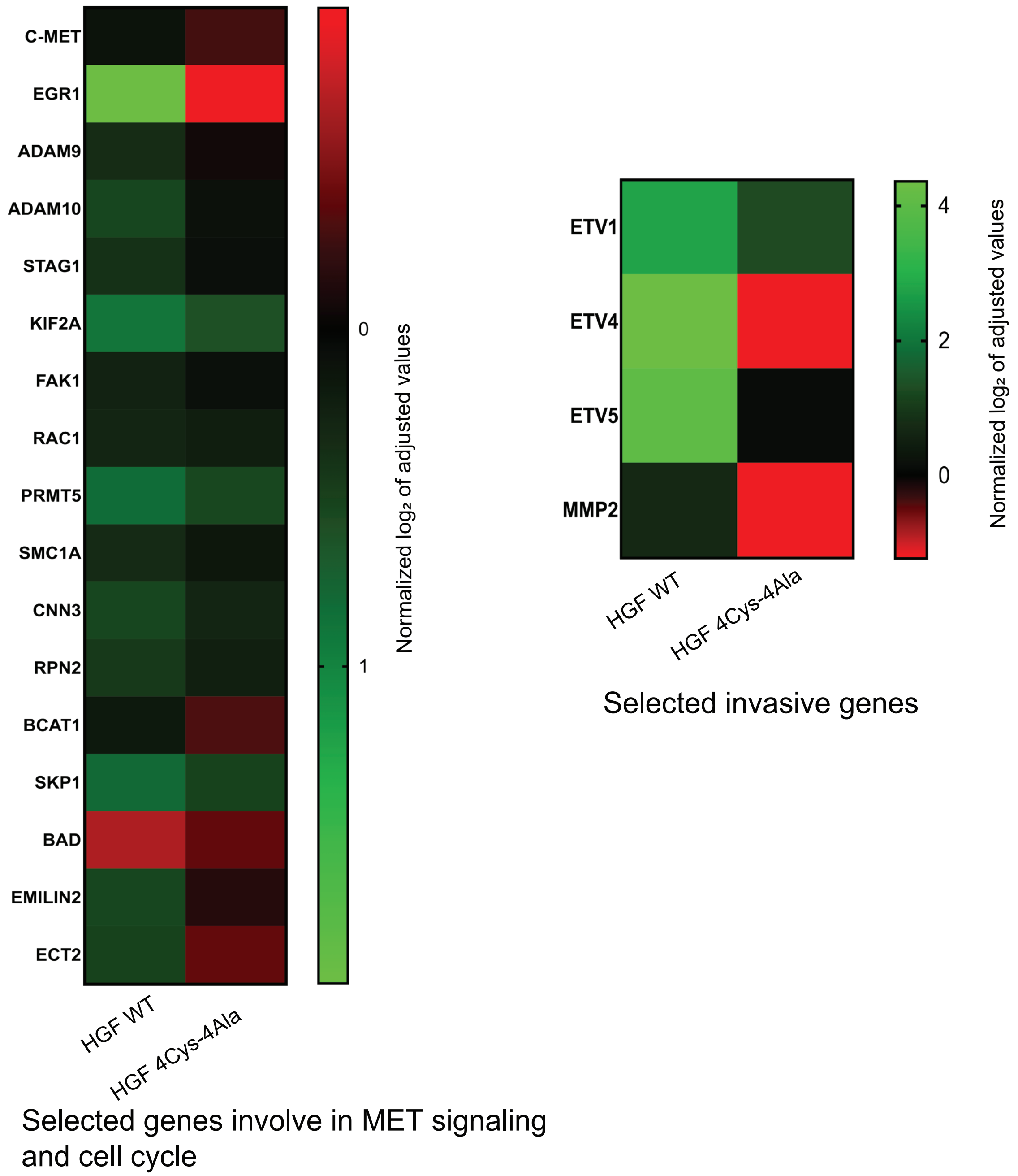

C-MET
EGR1
ADAM9
ADAM10
STAG1
KIF2A
FAK1
RAC1
PRMT5
SMC1A
CNN3
RPN2
BCAT1
SKP1
BAD
EMILIN2
ECT2
0
1
Normalized log₂ of adjusted values
HGF WT
HGF 4Cys-4Ala
Selected genes involve in MET signaling and cell cycle
ETV1
ETV4
ETV5
MMP2
4
2
0
Normalized log₂ of adjusted values
HGF WT
HGF 4Cys-4Ala
Selected invasive genes

Flag-HGF-WT

SDS-PAGE & Western blot Analysis:

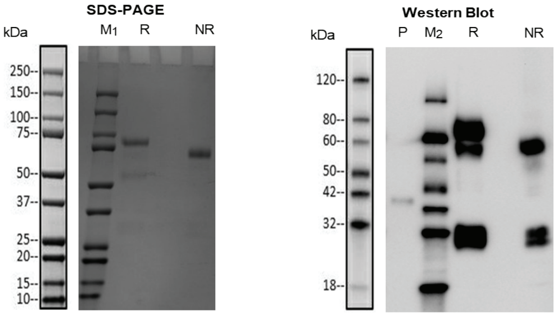

Lane M1: Protein Marker, Bio-rad, Cat. No. 1610374S, refer to annotated key on the left for size
Lane M2: Protein Marker, GenScript, Cat. No. M00673, refer to annotated key on the left for size
R: Reducing condition
NR: Non-reducing condition
Lane P: Multiple-tag (GenScript, Cat.No. M0101) as positive control
Primary antibody: Rabbit anti-FLAG pAb (GenScript, Cat.No. A00170)

Flag-HGF-C70A

SDS-PAGE & Western blot Analysis:

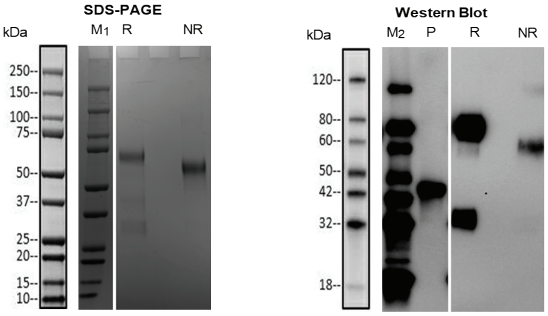

Lane M1: Protein Marker, Bio-rad, Cat. No. 1610374S, refer to annotated key on the left for size
Lane M2: Protein Marker, GenScript, Cat. No. M00673, refer to annotated key on the left for size
R: Reducing condition
NR: Non-reducing condition
Lane P: Multiple-tag (GenScript, Cat.No. M0101) as positive control
Primary antibody: Rabbit anti-FLAG pAb (GenScript, Cat.No. A00170)

Flag-HGF-C74A

SDS-PAGE & Western blot Analysis:

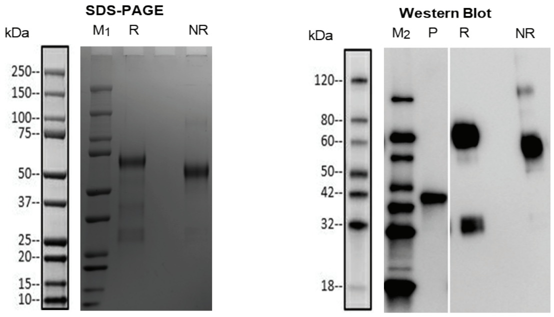

Lane M1: Protein Marker, Bio-rad, Cat. No. 1610374S, refer to annotated key on the left for size
Lane M2: Protein Marker, GenScript, Cat. No. M00673, refer to annotated key on the left for size
R: Reducing condition
NR: Non-reducing condition
Lane P: Multiple-tag (GenScript, Cat.No. M0101) as positive control
Primary antibody: Rabbit anti-FLAG pAb (GenScript, Cat.No. A00170)

Flag-HGF-C84A

SDS-PAGE & Western blot Analysis:

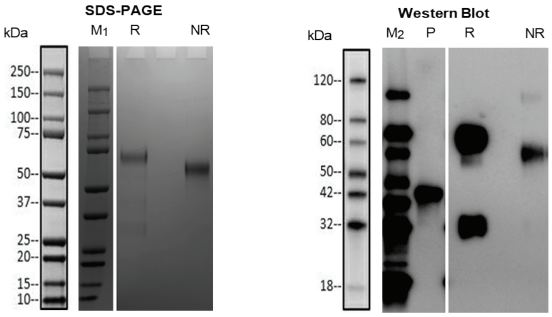

Lane M1: Protein Marker, Bio-rad, Cat. No. 1610374S, refer to annotated key on the left for size
Lane M2: Protein Marker, GenScript, Cat. No. M00673, refer to annotated key on the left for size
R: Reducing condition
NR: Non-reducing condition
Lane P: Multiple-tag (GenScript, Cat.No. M0101) as positive control
Primary antibody: Rabbit anti-FLAG pAb (GenScript, Cat.No. A00170)

Flag-HGF-C96A

SDS-PAGE & Western blot Analysis:

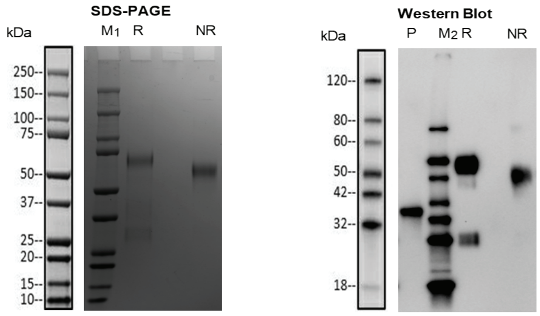

Lane M1: Protein Marker, Bio-rad, Cat. No. 1610374S, refer to annotated key on the left for size
Lane M2: Protein Marker, GenScript, Cat. No. M00673, refer to annotated key on the left for size
R: Reducing condition
NR: Non-reducing condition
Lane P: Multiple-tag (GenScript, Cat.No. M0101) as positive control
Primary antibody: Rabbit anti-FLAG pAb (GenScript, Cat.No. A00170)

Flag-HGF- 4C-4A

SDS-PAGE & Western Blot Analysis:

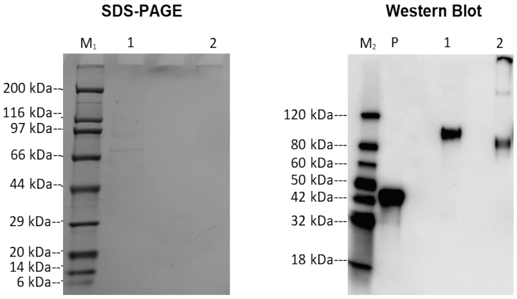

Fig.1 SDS-PAGE and Western blot analysis of 4 Cys to Ala HGF

Lane M1: Protein Marker, TaKaRa, Cat. No. 3452
Lane M2: Protein Marker, GenScript, Cat. No. M00521
Lane 1: Reducing condition
Lane 2: Non-reducing condition
Lane P: Multiple-tag (GenScript, Cat.No. M0101) as positive control
Primary antibody: Rabbit anti-FLAG pAb (GenScript, Cat.No. A00170)

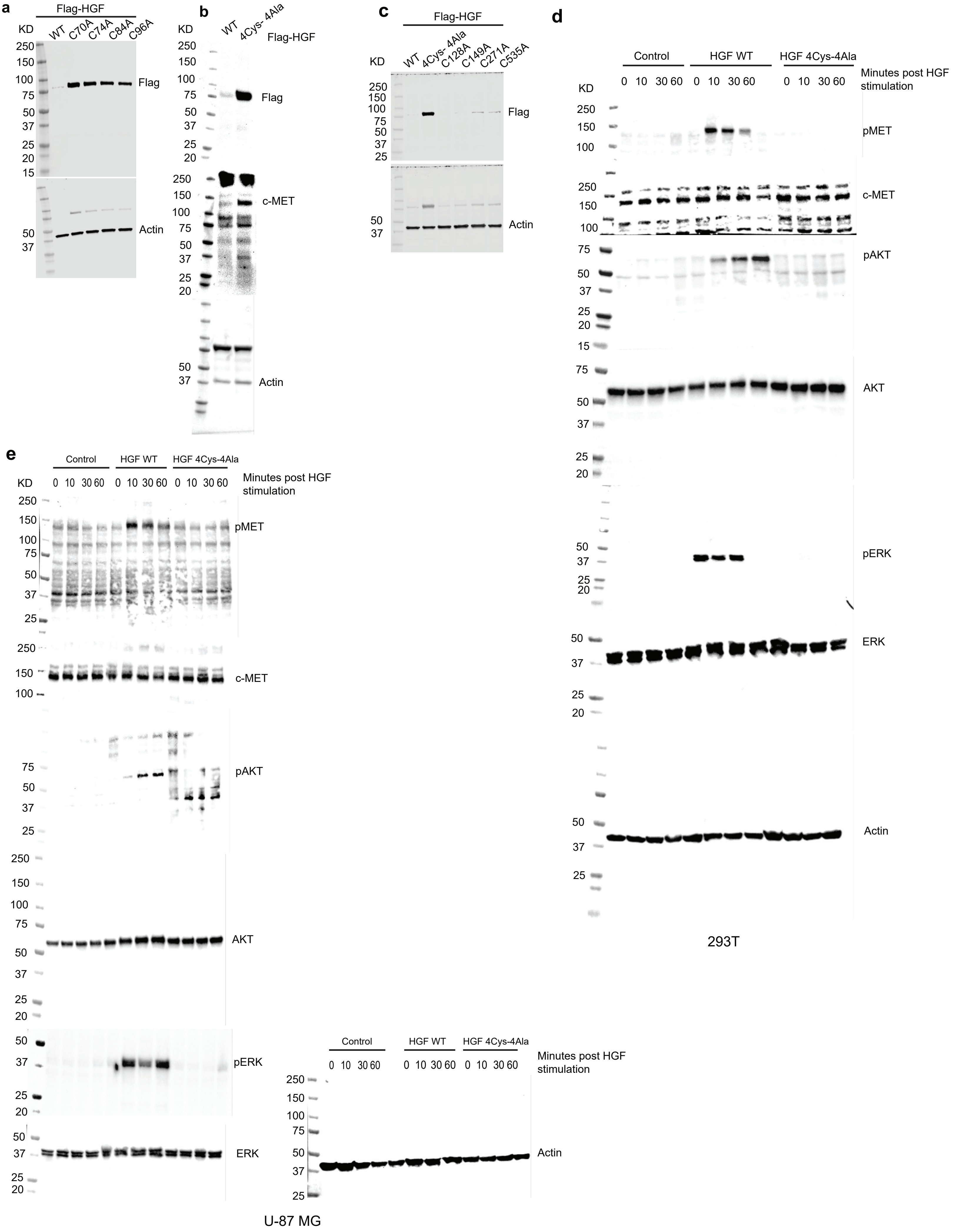
a
Flag-HGF
KD
WT
C70A
C74A
C84A
C96A
Flag
Actin
b
WT
4Cys- 4Ala
Flag-HGF
Flag
c-MET
Actin
c
Flag-HGF
WT
4Cys- 4Ala
C128A
C149A
C271A
C535A
Flag
Actin
d
Control
HGF WT
HGF 4Cys-4Ala
0 10 30 60
Minutes post HGF stimulation
pMET
c-MET
pAKT
AKT
pERK
ERK
Actin
293T
e
Control
HGF WT
HGF 4Cys-4Ala
0 10 30 60
Minutes post HGF stimulation
pMET
c-MET
pAKT
AKT
pERK
ERK
Actin
U-87 MG

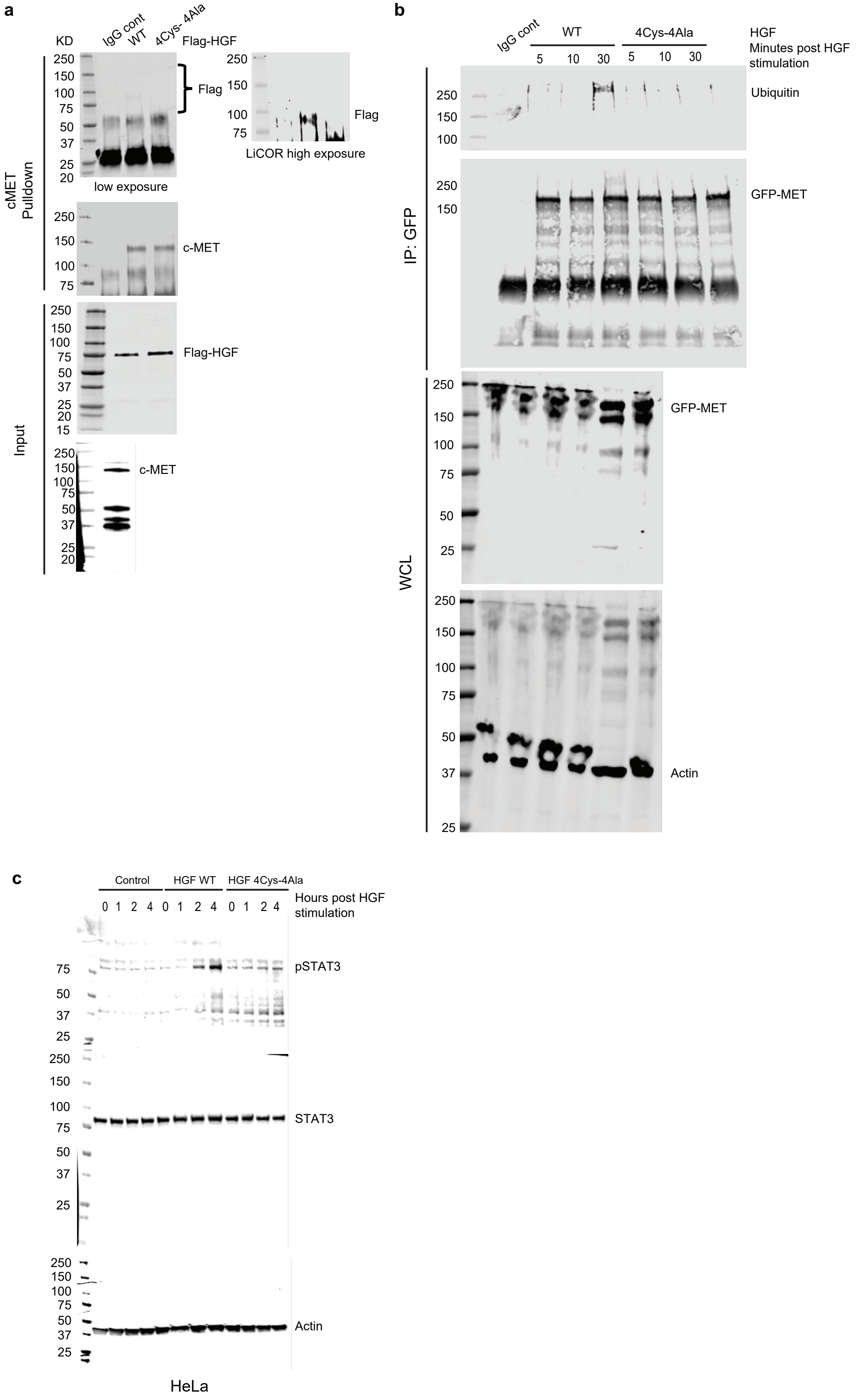
a
KD
IgG cont
WT
4Cys- 4Ala
Flag-HGF
Flag
LiCOR high exposure
low exposure
cMET Pulldown
c-MET
Input
Flag-HGF
c-MET
b
IgG cont
WT
4Cys-4Ala
HGF
Minutes post HGF stimulation
5
10
30
Ubiquitin
GFP-MET
IP: GFP
WCL
GFP-MET
Actin
c
Control
HGF WT
HGF 4Cys-4Ala
Hours post HGF stimulation
0 1 2 4 0 1 2 4 0 1 2 4
pSTAT3
STAT3
Actin
HeLa

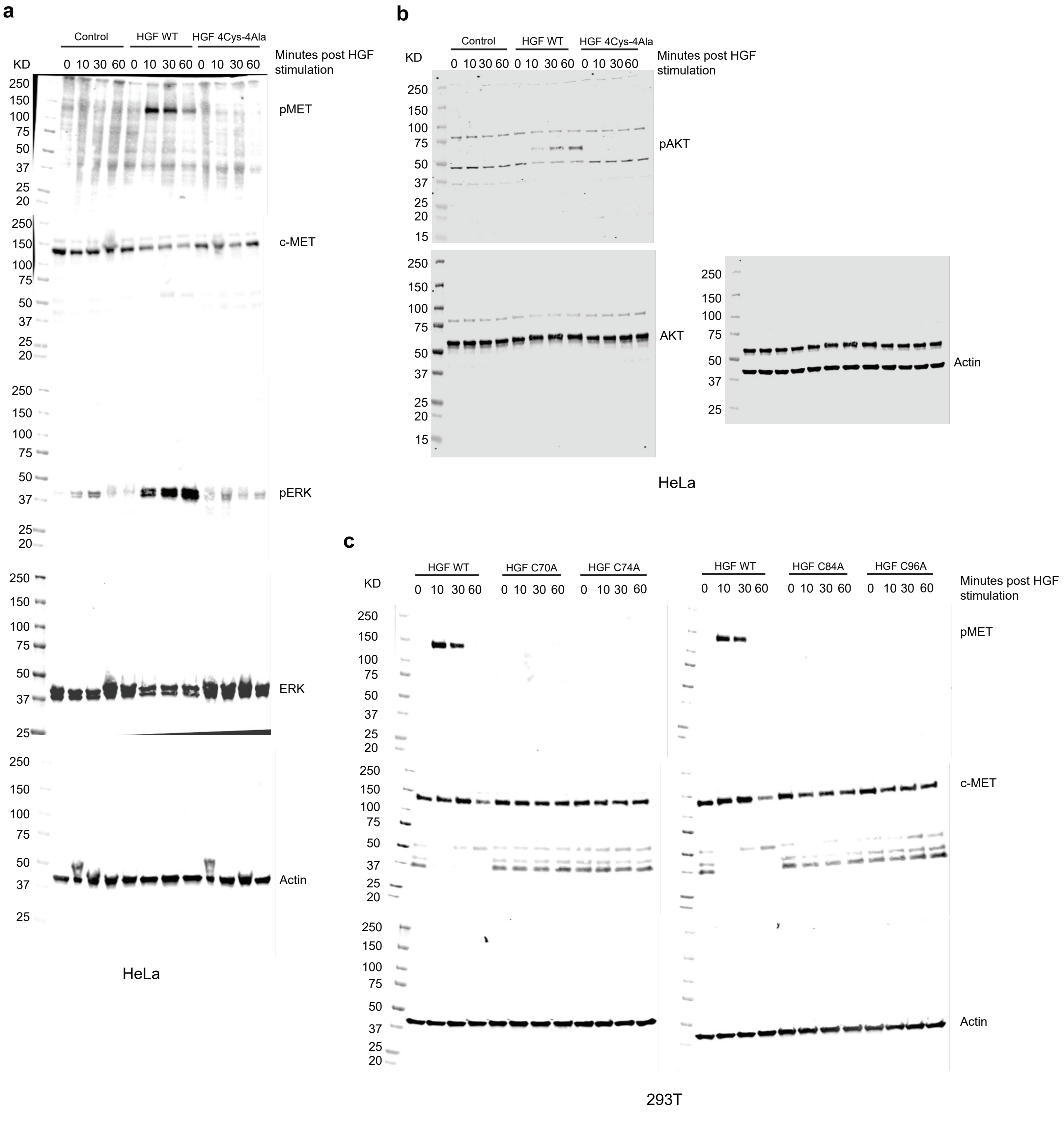
a
Control
HGF WT
HGF 4Cys-4Ala
KD
0 10 30 60 0 10 30 60 0 10 30 60
Minutes post HGF stimulation
250
150
100
75
50
37
25
20
pMET
c-MET
pERK
ERK
Actin
HeLa
b
Control
HGF WT
HGF 4Cys-4Ala
KD
0 10 30 60 0 10 30 60 0 10 30 60
Minutes post HGF stimulation
15
pAKT
AKT
Actin
HeLa
c
HGF WT
HGF C70A
HGF C74A
HGF WT
HGF C84A
HGF C96A
KD
0 10 30 60 0 10 30 60 0 10 30 60
Minutes post HGF stimulation
pMET
c-MET
Actin
293T

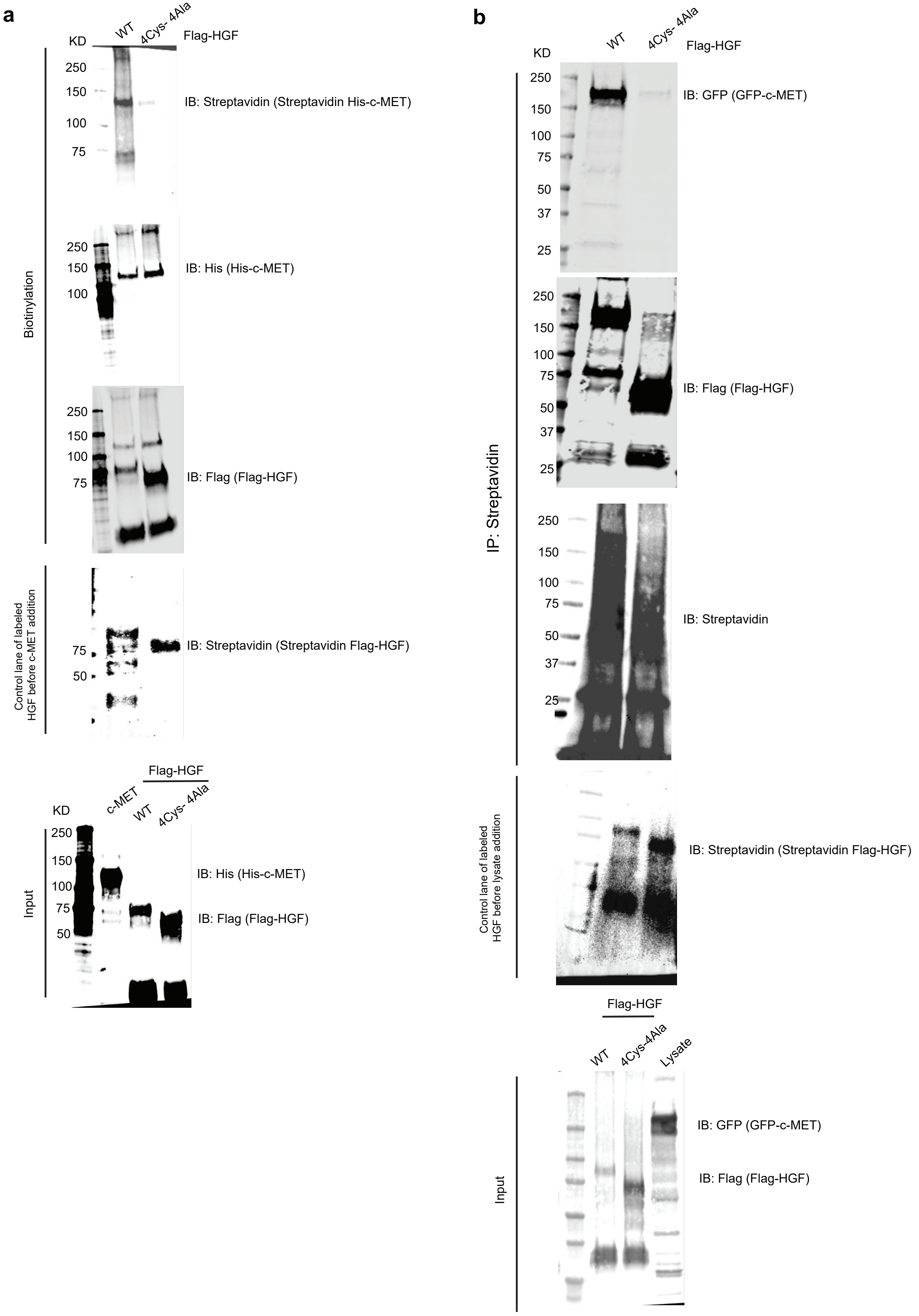
a
KD
WT
4Cys- 4Ala
Flag-HGF
250
150
100
75
IB: Streptavidin (Streptavidin His-c-MET)
250
150
100
IB: His (His-c-MET)
Biotinylation
250
150
100
75
IB: Flag (Flag-HGF)
Control lane of labeled HGF before c-MET addition
75
50
IB: Streptavidin (Streptavidin Flag-HGF)
Flag-HGF
KD
c-MET
WT
4Cys- 4Ala
250
150
100
75
50
IB: His (His-c-MET)
IB: Flag (Flag-HGF)
Input
b
KD
WT
4Cys- 4Ala
Flag-HGF
250
150
100
75
50
37
25
IB: GFP (GFP-c-MET)
250
150
100
75
50
37
25
IB: Flag (Flag-HGF)
IP: Streptavidin
250
150
100
75
50
37
25
IB: Streptavidin
Control lane of labeled HGF before lysate addition
IB: Streptavidin (Streptavidin Flag-HGF)
Flag-HGF
WT
4Cys-4Ala
Lysate
IB: GFP (GFP-c-MET)
IB: Flag (Flag-HGF)
Input

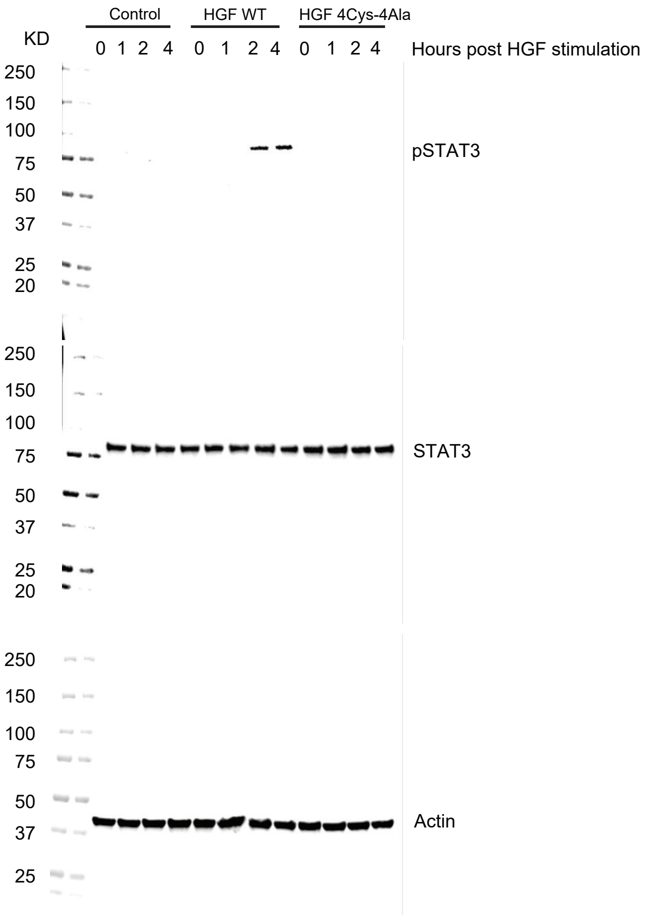
Control
HGF WT
HGF 4Cys-4Ala
KD
0 1 2 4 0 1 2 4 0 1 2 4
Hours post HGF stimulation
250
150
100
75
50
37
25
20
pSTAT3
250
150
100
75
50
37
25
20
STAT3
250
150
100
75
50
37
25
Actin

HeLa
