# Supplementary material for: Core cysteine residues in the Plasminogen-Apple-Nematode (PAN) domain are critical for HGF/c-MET signaling
Source: Commun Biol. 2022 Jul 1;5:646. doi: 10.1038/s42003-022-03582-8 (PMC9249922; doi:10.1038/s42003-022-03582-8)
Supplement: Supplementary file 2 — Description of Additional Supplementary Files [file 42003_2022_3582_MOESM2_ESM.pdf]

Description of Additional Supplementary Files

File name: Supplementary Data 1
Description: Species of origin, protein identification number (ID) and functional annotation of PAN domaincontaining proteins.

File name: Supplementary Data 2
Description: GO terms exhibiting significant enrichment among 28,300 PAN domain-containing proteins.

File name: Supplementary Data 3
Description: Proteins with PAN domain in alignment.
