# Supplementary material for: Core cysteine residues in the Plasminogen-Apple-Nematode (PAN) domain are critical for HGF/c-MET signaling
Source: Commun Biol. 2022 Jul 1;5:646. doi: 10.1038/s42003-022-03582-8 (PMC9249922; doi:10.1038/s42003-022-03582-8)
Supplement: Supplementary file 6 — Reporting Summary [file 42003_2022_3582_MOESM6_ESM.pdf]

nature research

Corresponding author(s): Wellington Muchero

Last updated by author(s): 05-31-2022

Reporting Summary

Nature Research wishes to improve the reproducibility of the work that we publish. This form provides structure for consistency and transparency in reporting. For further information on Nature Research policies, see our Editorial Policies and the Editorial Policy Checklist.

Statistics

For all statistical analyses, confirm that the following items are present in the figure legend, table legend, main text, or Methods section.

n/a	Confirmed	
☐	☒	The exact sample size (*n*) for each experimental group/condition, given as a discrete number and unit of measurement
☐	☒	A statement on whether measurements were taken from distinct samples or whether the same sample was measured repeatedly
☐	☒	The statistical test(s) used AND whether they are one- or two-sided<br>*Only common tests should be described solely by name; describe more complex techniques in the Methods section.*
☒	☐	A description of all covariates tested
☐	☒	A description of any assumptions or corrections, such as tests of normality and adjustment for multiple comparisons
☐	☒	A full description of the statistical parameters including central tendency (e.g. means) or other basic estimates (e.g. regression coefficient) AND variation (e.g. standard deviation) or associated estimates of uncertainty (e.g. confidence intervals)
☒	☐	For null hypothesis testing, the test statistic (e.g. *F*, *t*, *r*) with confidence intervals, effect sizes, degrees of freedom and *P* value noted<br>*Give P values as exact values whenever suitable.*
☒	☐	For Bayesian analysis, information on the choice of priors and Markov chain Monte Carlo settings
☒	☐	For hierarchical and complex designs, identification of the appropriate level for tests and full reporting of outcomes
☒	☐	Estimates of effect sizes (e.g. Cohen's *d*, Pearson's *r*), indicating how they were calculated

Our web collection on statistics for biologists contains articles on many of the points above.

Software and code

Policy information about availability of computer code

Data collection	No software was used for data collection.
Data analysis	all graphs related to qPCR and RNAseq were made by Prism software. Band intensity for western blots were measured by ImageJ and Licor. Images were made by Adobe photoshop and Adobe illustrator.

For manuscripts utilizing custom algorithms or software that are central to the research but not yet described in published literature, software must be made available to editors and reviewers. We strongly encourage code deposition in a community repository (e.g. GitHub). See the Nature Research guidelines for submitting code & software for further information.

Data

Policy information about availability of data

All manuscripts must include a data availability statement. This statement should provide the following information, where applicable:

- Accession codes, unique identifiers, or web links for publicly available datasets
- A list of figures that have associated raw data
- A description of any restrictions on data availability

Genetic screening results are available in supplemental tables. accession code associated with RNAseq is mentioned in this paper. No restriction on the distribution of the data availability used in this work.

Field-specific reporting

Please select the one below that is the best fit for your research. If you are not sure, read the appropriate sections before making your selection.

☒ Life sciences ☐ Behavioural & social sciences ☐ Ecological, evolutionary & environmental sciences

For a reference copy of the document with all sections, see nature.com/documents/nr-reporting-summary-flat.pdf

Life sciences study design

All studies must disclose on these points even when the disclosure is negative.

Sample size	Cell lines were used. Number of biological replicates were mentioned in the manuscript.
Data exclusions	no data points were excluded.
Replication	Experimental data were reliably reproduced in multiple independent experiments. For all experiments our data represents atleast two independent assay that produced similar results. No of independent experiments were indicated in each figure legends.
Randomization	No randomization since we used three different cell lines.
Blinding	N/A

Reporting for specific materials, systems and methods

We require information from authors about some types of materials, experimental systems and methods used in many studies. Here, indicate whether each material, system or method listed is relevant to your study. If you are not sure if a list item applies to your research, read the appropriate section before selecting a response.

Materials & experimental systems

n/a	Involved in the study
☐	☒ Antibodies
☐	☒ Eukaryotic cell lines
☒	☐ Palaeontology and archaeology
☒	☐ Animals and other organisms
☒	☐ Human research participants
☒	☐ Clinical data
☒	☐ Dual use research of concern

Methods

n/a	Involved in the study
☒	☐ ChIP-seq
☒	☐ Flow cytometry
☒	☐ MRI-based neuroimaging

Antibodies

Antibodies used	HA antibody (HA.C5 #18181; 1:1000) were purchased from Abcam. Flag (#2368S; 1:1000), (Met (clone 25H2 #3127; 1:1000), Phospho-Met (Tyr 1234/1235) (clone D26 # 3077; 1:1000), Stat3 (clone 124H6 #9139; 1:1000), Phospho-Stat3 (Tyr 705) (clone D3H7 #9145; 1:1000), Akt (pan) (clone 40D4 #2920; 1:1000), Phospho Akt (Thr 308) (clone 244F9 #4056; 1:1000) , p44/42 MAPK (Erk1/2) (#9102; 1:1000) and Phospho-p44/42 MAPK (Erk1/2) (Thr202/Tyr204) (clone D13.14.4E) (#4370; 1:1000) were purchased from Cell signaling. M2 anti Flag Mouse antibody (#SLBT7654; 1:5000) and Actin (#087M4850; 1:10,000) were purchased from Sigma. HA (#902302; 1:1000) antibody was purchased from Biolegend.
Validation	Commercial antibodies were used which are validated in manufacture website.

Eukaryotic cell lines

Policy information about cell lines

Cell line source(s)	ATCC
Authentication	Cell lines were obtained from ATCC and maintained under recommended conditions.
Mycoplasma contamination	Tested negative.
Commonly misidentified lines (See ICLAC register)	NO lines were used in this study.
